# High-Resolution Functional Profiling of Hepatitis C Virus Genome

**DOI:** 10.1371/journal.ppat.1000182

**Published:** 2008-10-17

**Authors:** Vaithilingaraja Arumugaswami, Roland Remenyi, Vidhya Kanagavel, Eric Yiang Sue, Tuyet Ngoc Ho, Chang Liu, Vanessa Fontanes, Asim Dasgupta, Ren Sun

**Affiliations:** 1 Department of Molecular and Medical Pharmacology, David Geffen School of Medicine, University of California, Los Angeles, California, United States of America; 2 Department of Microbiology, Immunology, and Molecular Genetics, David Geffen School of Medicine, University of California, Los Angeles, California, United States of America; 3 AIDS Institute, University of California, Los Angeles, California, United States of America; 4 Jonsson Comprehensive Cancer Center, University of California, Los Angeles, California, United States of America; 5 Molecular Biology Institute, University of California, Los Angeles, California, United States of America; University of California San Francisco, United States of America

## Abstract

Hepatitis C virus is a leading cause of human liver disease worldwide. Recent discovery of the JFH-1 isolate, capable of infecting cell culture, opens new avenues for studying HCV replication. We describe the development of a high-throughput, quantitative, genome-scale, mutational analysis system to study the HCV *cis*-elements and protein domains that are essential for virus replication. An HCV library with 15-nucleotide random insertions was passaged in cell culture to examine the effect of insertions at each genome location by insertion-specific fluorescent-PCR profiling. Of 2399 insertions identified in 9517 nucleotides of the genome, 374, 111, and 1914 were tolerated, attenuating, and lethal, respectively, for virus replication. Besides identifying novel functional domains, this approach confirmed other functional domains consistent with previous studies. The results were validated by testing several individual mutant viruses. Furthermore, analysis of the 3′ non-translated variable region revealed a spacer role in virus replication, demonstrating the utility of this approach for functional discovery. The high-resolution functional profiling of HCV domains lays the foundation for further mechanistic studies and presents new therapeutic targets as well as topological information for designing vaccine candidates.

## Author Summary

Hepatitis C virus (HCV) is a major human health concern that causes fatal liver diseases. Currently no vaccine is available to prevent HCV infection. Though the HCV was identified two decades ago, the virus has only recently been successfully grown in cell culture conditions. The role of HCV protein and regulatory element sub-domains during virus growth is poorly understood. We have developed a mutational analysis method to identify the function of HCV sub-domains at a high resolution. A collection of HCV mutants containing 15-nucleotide random insertions was tested for growth in cell culture. The precise location of the insertions and their effects on virus growth were analyzed by capillary genotyping technology and bioinformatics. Out of the total 2399 HCV mutants identified, 374 mutants grew normally, 111 mutants demonstrated reduced growth, and 1914 mutants failed to grow in cell culture. This mutational analysis method was validated by testing many individual mutant viruses. The present study identified several HCV functional sub-domains required for virus growth, presenting novel therapeutic targets. The HCV mutant viruses identified with the property of reduced growth can be used for designing vaccine candidates.

## Introduction

Hepatitis C virus (HCV) is a major human health concern with an estimated 130 million people infected with HCV worldwide [1] with resulting liver diseases including chronic hepatitis, cirrhosis, and hepatocellular carcinoma [2],[3]. Currently there is no effective vaccine, and the available treatment options offer limited response rates. HCV is classified in the family *Flaviviridae* and has a positive-sense, single-stranded RNA genome of about 9.6 kilobases (kb) [4]. The genome is organized as 5′NTR-C-E1-E2-p7-NS2-NS3-NS4A-NS4B-NS5A-NS5B-3′NTR, with non-translated regions (NTR) flanking a protein-coding region. The latter encodes a single polyprotein (∼3000 amino acids) that is co- and post-translationally cleaved by cellular and viral proteases into at least ten mature structural and non-structural (NS) proteins [5],[6],[7].

A recent review highlights the functions of HCV NTRs and proteins [8]. The 5′NTR *cis*-elements are involved in viral RNA translation and replication [9],[10],[11]. The core (C) and envelope glycoproteins form the structural proteins of the virion. The core nucleocapsid encapsidates the viral RNA genome [12],[13]. The ARF protein produced by a frame-shift translation of the core region is non-essential for virus replication [14],[15]. The envelope glycoproteins, E1 and E2, facilitate the virus entry into host cells through recognition of cellular receptors [16],[17]. An ion-channel forming peptide p7, and a cysteine protease NS2 play important roles in virion morphogenesis [18],[19],[20]. NS- 3, 4A, 4B, 5A and 5B form the replicase complex involved in RNA genome replication [21],[22],[23],[24]. The 3′NTR region is critical for initiation of negative-strand genome replication and translation enhancement [25],[26],[27]. To complete viral replication and transmission, the HCV proteins and *cis*-elements interact with various cellular factors, modulating signaling pathways and immune responses [28],[29],[30],[31].

The HCV sub-genomic replicon and chimpanzee infection model have previously been used for studying HCV replication [21],[24],[27]. Efficient replication of a genotype (GT) 2a HCV isolate JFH-1 in cell culture [32],[33],[34] offers the possibility for functional analysis of HCV proteins and NTRs. Despite these advances, the role of many HCV protein and *cis*-element sub-domains during infection remains unknown. Examining the function of viral factors by generating and testing individual mutant viruses would be time-consuming and labor-intensive for genome-scale studies. We have developed and applied a high-throughput mutational analysis approach to study the role of viral *cis*-acting elements and protein-domains in HCV replication utilizing transposon mutagenesis. Transposons have been used widely as a tool for studying the function of bacterial, yeast and viral genes [35],[36],[37]. For example, the Mu-transposon mediated 15-nucleotide (nt) insertion mutagenesis was used for mapping genomic regions crucial for propagation of *Potato virus* A [38], the 5′ end of human immunodeficiency virus type 1 [39], and pBC-SK plasmid [36]. These studies employed urea-polyacrylamide gel-based foot-printing to identify the insertion locations. We have developed a more rapid mutational analysis platform by integrating Mu-mediated random insertional mutagenesis and quantitative, high-throughput capillary electrophoresis genetic profiling. Using this platform, we have obtained a high-resolution functional profile of protein-domains and *cis*-acting regulatory elements that are critical for JFH-1 HCV replication in cell culture.

## Results

### Generation of a 15-nt insertion mutant JFH-1 plasmid library

**Figure 1:**
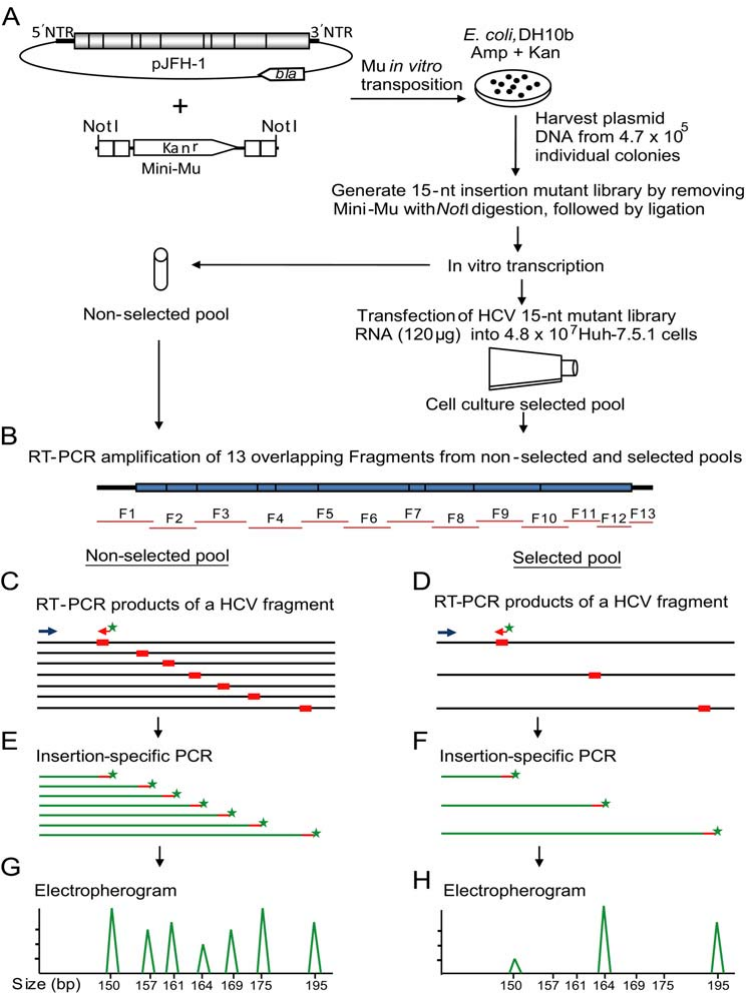
Schematic diagram depicting the various steps involved in Hepatitis C virus functional profiling. (A) The plasmid carrying the JFH-1 HCV genome is subjected to *in vitro* mutagenesis by using the mini-Mu transposon, then selected in *E. coli* bacteria. The harvested mutant plasmids are subjected to *Not*I restriction enzyme digestion to remove the transposon body, followed by ligation resulting in the generation of a 15-nt insertion plasmid library. Subsequently, this mutant plasmid library is *in vitro* transcribed and used as a non-selected input pool for functional profiling analysis. The *in vitro* transcribed RNA library is delivered into Huh-7.5.1 cells for genetic selection. The total RNA harvested from the transfected cells (selected pool) as well as non-selected pool RNA are subjected to functional profiling analysis. (B) Followed by selection, the mutant HCV genome from the non-selected input pool and the selected pool are reverse-transcribed and 13 overlapping fragments (F1 to F13) are PCR amplified. (C, D) The purified PCR products from non-selected and selected pools are used as templates for a second PCR using one of the HCV fragment-specific primers (blue arrow) and a fluorescently labeled insertion-specific primer (red arrow with green star). (E, F) The fluorescently-labeled PCR products from input and selected pools are analyzed by a 96-capillary genotyper. The processed data are either visualized by electropherograms (G, H) or exported as a data file. The phenotype for each insertion is calculated by comparing the corresponding peak areas of selected and non-selected pools.

A library of JFH-1 HCV mutants containing 15-nt insertions was generated by Mu-transposon insertion mutagenesis and subsequent removal of the transposon fragment (Fig. 1A). This yielded insertions of the 15-nt sequence 5′-NNNNNTGCGGCCGCA-3′ (N: duplicated 5 nucleotides from target DNA), coding for different amino acid sequences depending on the insertional reading frame (Fig. S1). The 15-nt insertion does not introduce a stop codon [38]. Insertion mutations were used for analyzing the structure-function of the proteins [38],[39],[40]. Sequencing of random clones indicated that 82% (27/33) of transposon insertions were widely distributed in the JFH-1 genome and 18% (6/33) of the insertions were in vector sequences.

### Genetic selection and analysis method of mutant JFH-1 library

The *in vitro* transcribed JFH-1 library was used as a non-selected RNA input pool for functional profiling analysis and for genetic selection for growth in cell culture (Fig. 1). To identify HCV protein domains and *cis*-acting elements critical for virus replication, the mutant RNA library was passaged en masse in Huh-7.5.1 cells. At 21 days post-transfection (dpt), most of the cells showed cytopathic effects (CPE) as previously reported [34],[41]. The kinetics of mutant library genome replication and viral titer are shown in Fig. S2. The cDNA generated from the harvested total RNA was used as a template for functional profiling PCR. A total of thirteen overlapping HCV fragments (F1 to F13) were PCR-amplified from the cDNA of the non-selected and cell culture-selected mutant libraries (Fig. 1B and Table S1). Subsequently, the purified PCR products were used as templates for a second PCR using an insertion-specific fluorescent-labeled primer and one of the forty-eight JFH-1 specific primers (Fig. 1C–F and Table S2), and these fluorescent-labeled PCR products were analyzed by capillary electrophoresis (Fig. 1G,H).

**Figure 2:**
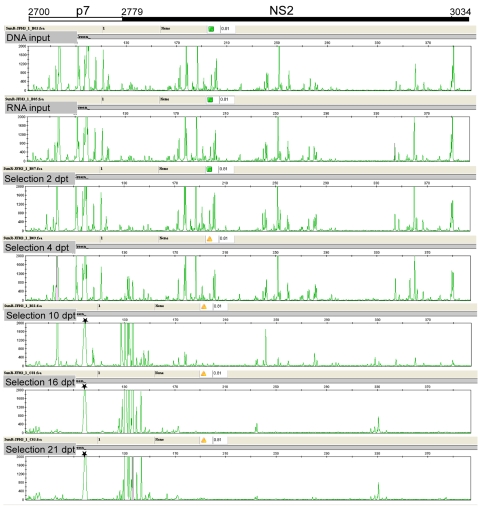
Electropherogram depicting the location of 15-nt insertions in p7-NS2 region and the mutant population replication dynamics during selection. Each peak (X-axis) represents the location of a 15-nt insertion in the p7-NS2 region, and the fluorescent signal intensity (Y-axis) indicates the abundance of each 15-nt insertion mutant. The number at the top of the figure corresponds to the JFH-1 genome position of the p7-NS2 region. The electropherogram panels show the insertion profile of the mutant plasmid library (DNA input), *in vitro* transcribed RNA library (RNA input), and Huh-7.5.1 cell culture selected mutant viral library [selection 2, 4, 10, 16, and 21 days post-transfection (dpt)]. The complexity of the library is similar in DNA input, RNA input, 2 dpt and 4 dpt. Most of the 15-nt insertion mutants have been negatively selected at 10 dpt. Note that the HCV mutants containing 15-nt insertions around p7-NS2 junctions have shown strong positive selection. Asterisks indicate an artifact peak generated during data processing. To better visualize the short peaks, the fluorescent signal intensity scale was set at 2000; hence some of the tall peaks are shown out of scale.

To ascertain that the complexity of the mutant library was maintained during *in vitro* transcription and transfection, the JFH-1 plasmid library, the *in vitro* transcribed RNA library, and the total cellular RNA harvested at 2, 4, 10, 16 and 21 days post-transfection were subjected to functional profiling analysis. Analysis of the p7-NS2 region showed that the complexity of the library was maintained through *in vitro* transcription and during the early phase of selection in Huh-7.5.1 cells (Fig. 2). At 10 dpt many of the insertion mutants had been negatively selected, and by 21 dpt only a limited number of clones had continued to replicate. By comparison, all insertions at the 3′NTR poly(U) tract were negatively selected by 2 dpt (Fig. S3), confirming its critical role in viral genome replication [25],[26],[27],[42]. Thus, the functional profiling system could be useful for monitoring the replication kinetics of individual insertion mutants.

### Functional profile of the HCV genome

**Figure 3:**
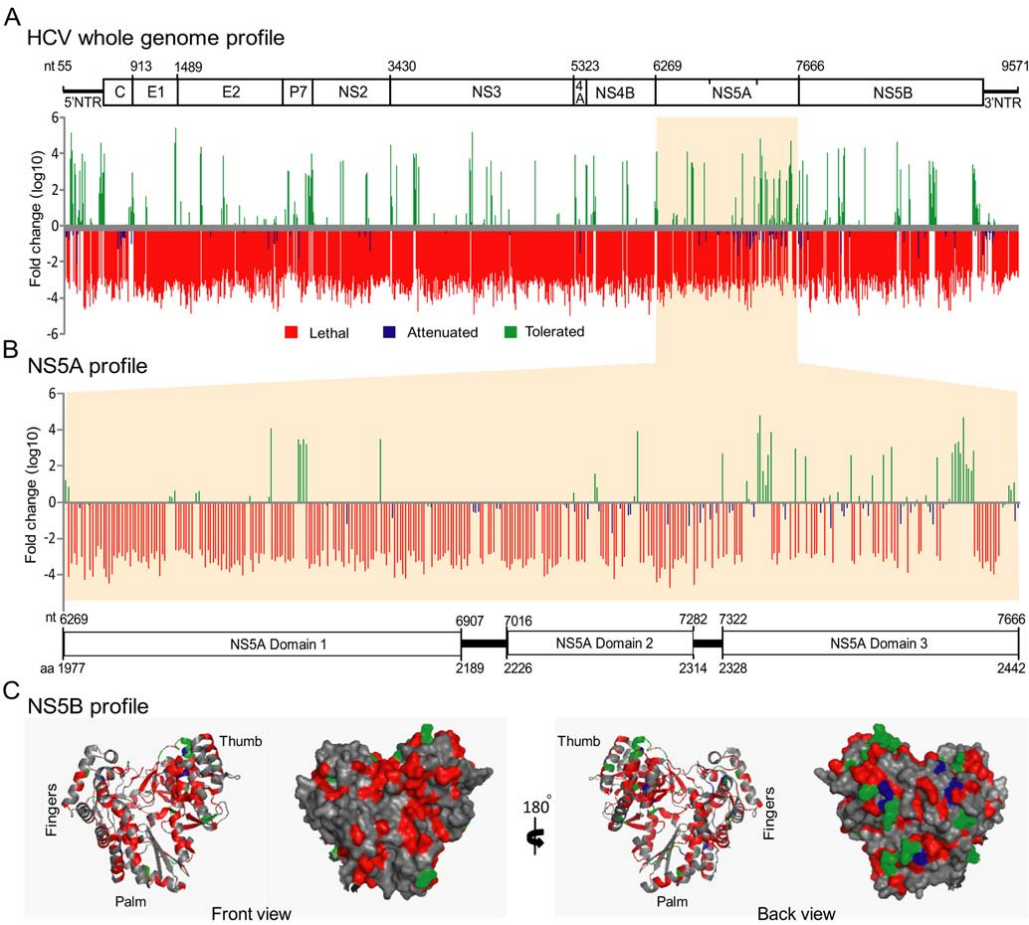
Genome scale functional profile of HCV. Graphical representation of the location and phenotype of 15-nt insertions in the HCV genome are shown. For each 15-nt insertion mutant, the ratio of peak area was calculated between selected and non-selected pools and plotted in a bar graph. The lethal phenotype (critical region, red bar) is an absence of an insertion mutant in the selected population. The attenuated phenotype (less critical region, blue bar) is an over two-fold reduction in replication. The tolerated phenotype (dispensable region, green bar) is replication competent. (A) The final assembly shows the fold change (log_10_) and locations of insertions in the HCV genome. A cartoon of the HCV regions is aligned at the top of graph to show the boundary of each region. The numbering corresponds to the nucleotide (nt) position of JFH-1 genome. (B) The location and phenotype of insertions at the NS5A region are shown. A schematic diagram of the NS5A domains is aligned with the functional profile graph. Note that many insertions at domain 3 are tolerated. (C) The crystal structure of NS5B [69] (PDB accession code 1C2P), RNA dependent RNA polymerase, displays the functional profiling phenotypes. The front and back views of ribbon and surface diagrams of the NS5B monomer is shown. The fingers, thumb, and palm sub-domains are indicated. The amino acid residues are color coded for insertion phenotypes: red (lethal), blue (attenuating), green (tolerated), and grey (no insertion). Insertions in the sub-domains forming the catalytic active site were lethal (front view) for virus replication, whereas many insertions on the outer surface (back view) were tolerated. The crystal structure was analyzed using PyMOL Viewer. aa, amino acid.

*In vitro* genetic selection resulted in maintenance (neutral or tolerated fitness), loss (lethal fitness), or reduction (attenuated fitness) of individual insertion mutants over time. To define a phenotype for each insertion mutant, the ratio of peak area between selected (21 dpt) and non-selected pools was calculated. The insertion resulting in absence, two fold reduction, or maintenance was assigned a lethal, attenuated, or tolerated phenotype, respectively. In the present study, the phenotype attenuation indicates reduction in virus replication, not loss of virulence. The final assembly containing the locations of insertion sites and corresponding phenotypic annotations for 2399 independent insertions across the entire HCV genome (nt 55 to 9571) (Fig. 3) was obtained. For a high-resolution insertion profile map see Fig. S4. The results showed that 79.8% (1914) of the insertions were lethal, 4.6% (111) attenuating, and 15.6% (374) tolerated, with respect to viral replication. The total number of insertions and their effect on virus replication for each of the HCV regions are shown in Table 1.

**Table 1:** The percentage and number of insertions in various regions of HCV.

Region	Genome Location	Size (nucleotides)	Total Insertions in a Region	Tolerated	Attenuated	Lethal
5′NTR	55–340	286	93 (3.9%)	37 (39.8%)	8 (8.6%)	48 (51.6%)
Core	341–913	573	81 (3.4%)	24 (29.6%)	8 (9.9%)	49 (60.6%)
E1	914–1489	576	109 (4.5%)	6 (5.5%)	0 (0.0%)	103 (94.5%)
E2	1490–2590	1101	272 (11.3%)	24 (8.8%)	8 (3.0%)	240 (88.2%)
p7	2591–2779	189	64 (2.7%)	18 (28.1%)	2 (3.1%)	44 (68.8%)
NS2	2780–3430	651	199 (8.3%)	15 (7.5%)	3 (1.5%)	181 (91.0%)
NS3	3431–5323	1893	467 (19.5%)	39 (8.4%)	5 (1.0%)	423 (90.6%)
NS4A	5324–5485	162	26 (1.0%)	3 (11.5%)	1 (3.9%)	22 (84.6%)
NS4B	5486–6268	783	177 (7.4%)	23 (13.0%)	0 (0.0%)	154 (87.0%)
5A Region 1	6269–7016	748	170 (7.1%)	21 (12.4%)	8 (4.7%)	141 (82.9%)
5A Region 2	7017–7318	302	82 (3.4%)	12 (14.6%)	18 (21.0%)	52 (63.4%)
5A Region 3	7319–7666	348	106 (4.4%)	48 (45.3%)	16 (15.1%)	42 (39.6%)
NS5B	7667–9442	1776	467 (19.5%)	92 (19.7%)	22 (4.7%)	353 (75.6%)
3′NTR	9443–9571	129	86 (3.6%)	12 (13.95%)	12 (13.95%)	62 (72.1%)
**Total**	55–9571	9517	2399 (100.0%)	374 (15.6%)	111 (4.6%)	1914 (79.8%)

Whole genome profiling demonstrated distinct patterns for each genetic region. Comparison of the genome-scale functional profile with known HCV sequence variability revealed that the conserved HCV proteins, including core, NS- 3, 4A, 4B, and 5A N-terminus, had fewer tolerated insertions. The less-conserved regions, including p7-NS2 junction and 5A C-terminus, had many tolerated insertions. An exception was the envelope proteins (E1 and E2), which have highly variable amino acid sequences but were intolerant for insertions.

#### Functional profile of 5′NTR

**Figure 4:**
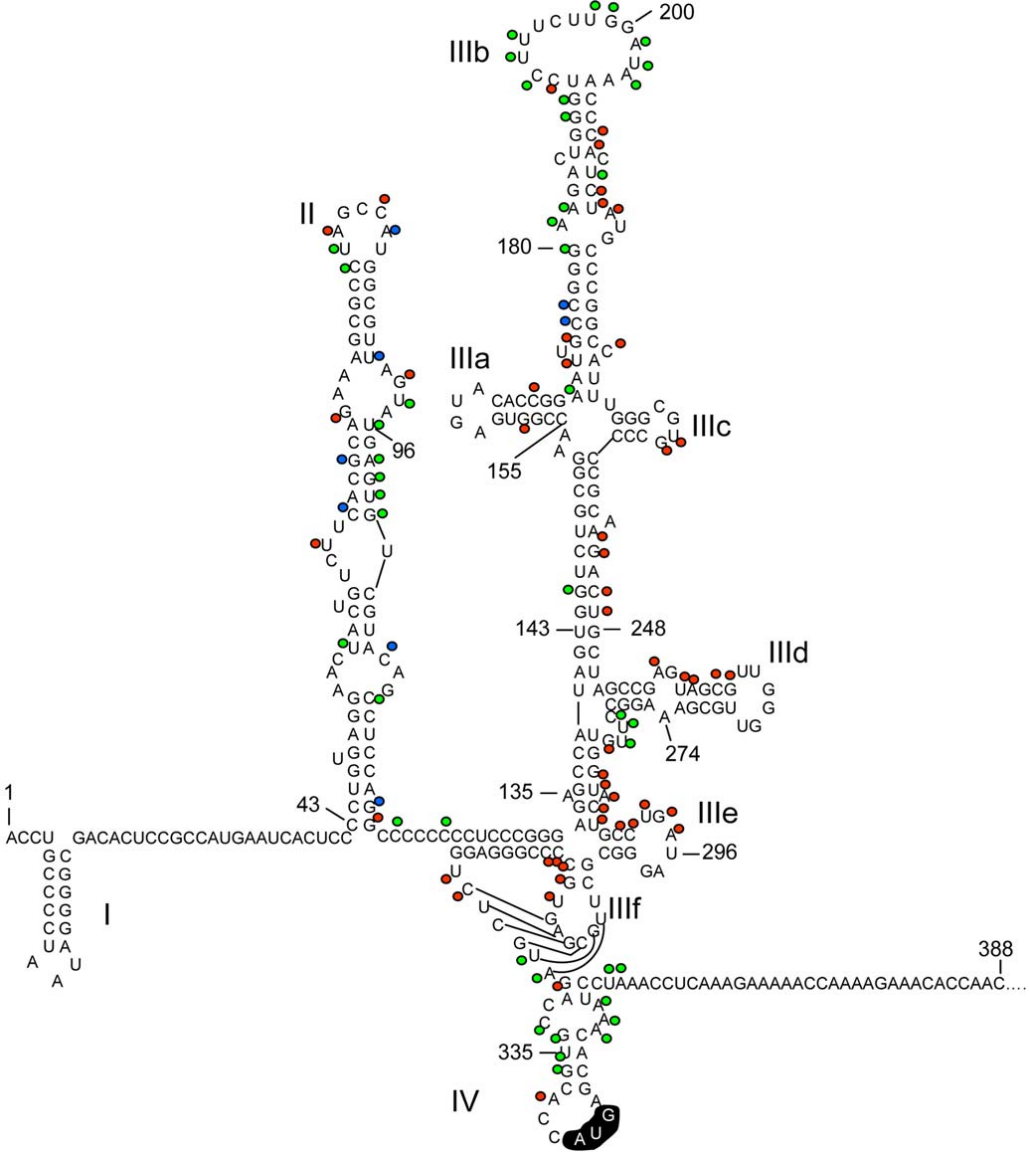
Functional Profiles of the JFH-1 HCV 5′NTR *cis*-elements. The predicted secondary structures of 5′NTR are shown. The numbers correspond to the JFH-1 genome sequence. The locations of 15-nt insertions are indicated as filled circles. The colors of the filled circles represent the phenotypes: lethal (red), attenuating (blue), and tolerated (green). 5′NTR stem loop domains (I, II, III and IV) are shown. The loop region of IIIb and stem loop domain IV had many tolerated insertions. The translation initiation codon AUG is highlighted.

5′NTR is a highly conserved region of HCV. The 5′NTR *cis*-elements are involved in viral RNA replication and translation, consisting of four major stem-loop (SL) structural domains: I, II, III, and IV (Fig. 4) [43]. SL III contains six additional minor SL structures. The stem-loops II, III, and IV constitute an internal ribosome entry site (IRES) which mediates cap-independent initiation of RNA translation. The 5′NTR is recognized by several cellular factors [28],[29]. The SL II domain consists of nucleotides 43–117. Majority of the 15-nt insertions between nt 62–93 were either lethal or attenuating (Fig. 4). Between nt 94–101, all the insertions were tolerated. Two insertions at the pyrimidine tract-I (Py-I), the region that connects SL II and III, were tolerated. All insertions in the SL III- a, c, d, and e and the IIIf pseudoknot were lethal for virus replication, which is consistent with their role in binding to the 40S ribosomal subunit [43]. The eIF3 contact sites have been mapped to SL IIIb and the junction of stems III- a, b, and c [43]. Most of the insertions at the IIIb apical loop (nt 180–203) containing the pyrimidine tract-II (Py-II) were tolerated for virus replication. The nucleotide sequence of Py-I and II regions have been shown to be non-essential for IRES-mediated translation [44]. Collier and colleagues [45] reported that the SL IIIb internal loop is critical for IRES activity than the apical loop which corroborates our mutational profile. Many of the insertions were tolerated at domain IV where the translation start codon, AUG, is located. An insertion at nt 338 was lethal for virus replication. A previous study has shown that the stability of the domain IV stem loop is negatively correlated to the initiation of translation [46]. Analysis of the predicted domain IV secondary structures with the 15-nt insertion revealed that the tolerated insertions maintain an open destabilized structure similar to that of wild-type domain IV, whereas the lethal insertion forms a stable hairpin structure (Fig. S5). Thus, the tolerated insertions at domain IV could allow translation-initiation, resulting in virus replication.

#### Functional profile of Core

The structural protein, core, encapsidates the genomic RNA in the virus particle. The core region functional profiling showed that the insertions at nt 349–356 (3–6 aa), which is also a part of the IRES, were tolerated. Most of the insertions (between aa 23–107) at the RNA binding basic domain were lethal. Insertions at the hydrophobic domain between aa 133–167 were mostly lethal and attenuating. Insertions found between aa 176–191 of the C-terminal transmembrane region were tolerated. The role of core protein in infectious virus particle production has been studied by alanine scanning mutagenesis [13]. Most of our data is consistent with the findings of that study, including the tolerated mutations in the transmembrane region. The reported study has shown that alanine substitutions of stretches of four residues at aa 173–176, 177–180 were lethal for infectious particle production. We have found, however, that the insertions at aa 176, 177, and 180 were tolerated. This finding could be explained by the fact that the insertion results in duplication of amino acids at the site of Mu-integration that preserves the original amino acid without deletion or substitution (Fig. S1).

#### Functional profiles of E1 and E2 proteins

The envelope glycoproteins, E1 and E2, facilitate virus entry into host cells through recognition of cellular receptors [16],[17],[47]. The 15-nt insertions in the E1 and E2 regions were mostly deleterious for virus replication. The percentage of lethal insertions at the E1 and E2 regions were 94.5 and 88.2, respectively (Table 1). The E1 N-terminal residues 244–245 and the C-terminal residues 370 and 373–374 had tolerated the insertions. Insertions at aa 481–482, 503–504, and 598, and 622–623 of E2 region were tolerated as well. It has been shown that the recognition of cellular receptors is dependent on the conformation of the envelope glycoproteins [16]. Thus the insertion could possibly affect the host receptor protein interactions, conformation of glycoproteins, and innate-immune evasion function, resulting in a lethal phenotype.

#### Functional profiles of p7 and NS2

The p7 is an ion channel-forming transmembrane protein. It has two transmembrane domains (TMD) with a single loop facing the cytosol and its N- and C-terminal tails oriented towards the endoplasmic reticulum lumen [48]. The insertions at the junction of the N-terminal tail and the first TMD (aa 760–765) were tolerated for virus replication. Insertions at the cytosol loop region aa 781 and aa 788–789 were tolerated. Four insertions found between aa 782–787 of the loop region were lethal. Most of the insertions at TMD regions were lethal. Many insertions at the p7-NS2 cleavage site, aa 809–818 (nt 2767–2794), were tolerated or positively selected (Figs. 2,3A). 91% of the insertions at NS2 were lethal for virus replication, underscoring the importance of NS2 in the HCV life cycle. The NS2 and NS3 crystal structures [20],[49] with the insertion profiles are shown in Fig. S6.

#### Functional profiles of NS3 and NS4A

The NS3 is a critical component of the RNA replicatory complex. NS3 is comprised of an N-terminal serine protease domain and a C-terminal RNA helicase/nucleoside triphosphatase domain [6],[50]. NS3 plays an important role in immune evasion [30],[31]. The NS3 protease domain forms a complex with NS4A that is essential for the processing of NS3/4A, NS4A/4B, and NS4B/5A cleavage sites [7],[51]. NS3 is a highly conserved region of HCV and not surprisingly, most of the insertions were lethal for virus replication. Insertions at both the N-terminal (aa 1031–1035) and the C-terminal (aa 1658–1660) ends were tolerated. Insertions at the protease domain aa 1126–1129 (Pro-Cys-Lys-Cys), a sub-domain containing cysteine residues which coordinates a zinc atom, were tolerated for virus replication (Fig. S6). Insertions at helicase domain aa 1304–1306 and aa 1312 were tolerated. In NS4A, one insertion at the membrane anchoring domain (aa 1675), and one at the C-terminal acidic domain (aa 1713) were tolerated. The remaining insertions were deleterious for virus replication.

#### Functional profile of NS4B

NS4B, a transmembrane protein, is essential for viral genome replication [21],[24],[52]. An amphipathic helix (AH) region present at the N-terminal is critical for viral RNA replication [53]. Consistent with that study, we found that insertions at the AH region (aa 1730–1745) were lethal for virus replication. It has been reported that the N- and C-terminal less-conserved domains were the major determinants for efficient RNA replication [54]. The insertions at the determinant N-terminal (aa 1754–1760) and C-terminal (aa 1974–1976) regions were tolerated. Insertions at the ends of a predicted cytoplasmic loop region [55] aa 1847–1848 and aa 1856–1857 were tolerated, however, insertions in the mid-loop aa 1849–1854 were deleterious. Insertions in all four predicted transmembrane domains were lethal. Many of the insertions at the cleavage sites of NS4B/5A and NS5A/5B were tolerated. The predicted amino acid sequences of the insertion sites revealed that the insertion did not disrupt the critical P1-P1′ cleavage residues (Fig. S7).

#### Functional profile of NS5A

The NS5A is essential for viral genome replication; however its precise role in the virus life cycle is unknown. NS5A is a membrane-bound, phosphorylated, zinc-binding metalloprotein [56],[57],[58]. It modulates the cellular environment by direct or indirect association with host factors [28],[59],[60]. Many cell culture adaptive mutations have been mapped to NS5A [24],[41]. NS5A is predicted to have three domains [61]. Our findings showed that 82.9% of the insertions at NS5A region-1, 63.4% at region-2, and 39.6% at region-3 were lethal (Fig. 3B and Table 1). Many insertions in region- 2 and 3 had an attenuating phenotype. Insertions at aa 2014–2015 (Cys-residue binding to zinc) were tolerated and the predicted insertion sequence showed that the insertion did not affect the Cys residue involved in zinc binding. The NS5A crystal structure [56] with the mutational profile is shown in Fig. S6. Insertions in the region aa 2209–2254, corresponding to a 47 aa deletion, encompassing the interferon sensitivity determining region [60], that was tolerated for sub-genomic RNA replication [24], had severely impaired virus replication fitness. All but one of 22 insertions between aa 2282–2320 of region-2 were deleterious for virus replication, which further supports the essential role of these residues in sub-genomic HCV RNA replication [62]. Our profiling analysis showed that the NS5A region-3 was the most tolerated (48%) region for insertion. Previous studies using sub-genomic replicons have shown that the NS5A C-terminal had tolerated heterologous insertions, including green fluorescent protein (GFP) and transposons [58],[63],[64], however a sub-genomic mutant with a larger insert (*Renilla* luciferase gene) had a defect in viral RNA replication [58]. A recent report showed that the GFP insertion at the NS5A C-terminal resulted in over 100-fold reduction in infectious virus production, but had no effect on viral RNA replication [65]. It has been shown that the C-terminal serine residue (aa 2433) is critical for infectious virus production [66]. We have found that insertions between aa 2429–2437 were lethal for virus replication. Through characterizing mutant viruses having serial deletions of domain III, a report has shown that the NS5A domain 3 is involved in the assembly of infectious viruses [67]. These mutant viruses exhibited phenotypes ranging from defective to normal growth properties. In addition to tolerated insertions, a total of 39.6% of the insertions at region-3 resulted in a lethal phenotype, suggesting that this region plays a critical role in viral replication fitness.

#### Functional profiles of NS5B and *cis*-element 5BSL3

**Figure 5:**
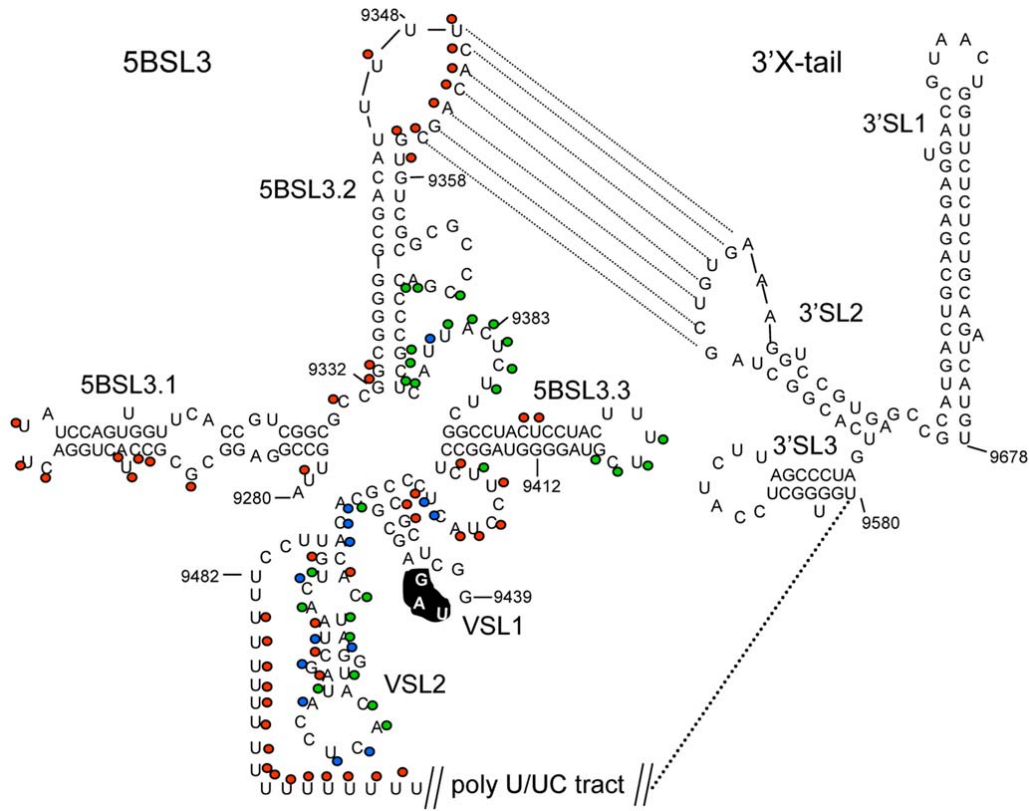
Functional Profiles of the JFH-1 HCV 5BSL3 CRE and 3′NTR *cis*-elements. The stem loop structures are shown. The colors of the filled circles represent the phenotypes: lethal (red), attenuating (blue), and tolerated (green). Insertions at 5BSL- 3.1 and 3.2 were lethal for virus replication. Many insertions at the bulge region between CRE 5BSL- 3.2 and 3.3 were tolerated. The kissing-loop interaction between CRE 5BSL3.2, and 3′SL2 is depicted with dotted lines. The 3′NTR predicted variable region stem loop structures (VSL1 and VSL2), poly (U/UC) tract, and 3′X tail stem loop structures are shown. Many insertions at VSL2 were tolerated and attenuating. The stop codon UAG is highlighted. All the insertions at poly-U tract were lethal.

NS5B is an RNA-dependent RNA polymerase that consists of fingers, palm, and thumb domains involving polymerase activity, and C-terminal regulatory and membrane-anchoring domains [68],[69],[70]. Analysis of functional profiles incorporated in the crystal structure of NS5B [69] showed that all of the insertions in the sub-domains forming the catalytic site were lethal, whereas several insertions on the outer surface were tolerated (Fig. 3C). 75.6% of insertions in NS5B had a deleterious effect and 19.7% insertions had no effect on virus replication. Insertions at a loop region (aa 2589–2592) that interconnects fingers and thumb domains, were tolerated. The palm domain loop region aa 2793–2795 and 2797–2798 tolerated the insertions. Tolerated insertions were found at aa 2814–2816 of the junction of the palm-thumb domains. Insertions at the thumb domain helix (aa 2873–2881) were well tolerated for virus replication. Insertions at the C-terminal transmembrane region aa 3012–3016 (nt 9368–9386) were tolerated, while insertions at aa 3027–3031(nt 9421–9433) were deleterious (Figs. S3,S4). The NS5B coding region contains a *cis*-acting replication element (CRE) predicted to have a cruciform stem loop structure, 5BSL3 [71],[72] (Fig. 5). The formation of a kissing-loop interaction between loop regions of 5BSL3.2 and 3′NTR SL2 is critical for viral RNA replication [26],[72]. Our mutational profile showed that the insertions at 5BSL3.1 (nt 9291–9357) and 5BSL3.2 were lethal for virus replication. All but one of 14 insertions at nt 9368–9386, which encompass a bulge region between 5BSL3.2 and 5BSL 3.3, were tolerated. The insertions at this region could affect the function of both the NS5B protein and the 5BSL3 *cis*-element.

#### Functional profile of 3′NTR *cis*-elements

3′ NTR consists of a proximal variable region (VR), poly(U/UC) tract of varying length, and a conserved 3′X tail [27] (Fig. 5). The 3′NTR region is involved in initiation of negative-strand genome replication and enhancement of translation [25],[26],[27]. Several cellular and viral proteins interact with 3′NTR [28],[73]. The functional profile of the variable region and poly (U/UC) tract was obtained. Among a total of 27 insertions at the 3′NTR variable region *cis*-element, 11, 11, and 5 of the insertions had tolerated, attenuating, and lethal phenotypes, respectively. Four of the 5 insertions exhibiting lethal phenotypes at VR were found adjacent to the poly(U/UC) tract. All of the insertions at the poly(U/UC) tract were lethal for virus replication (Fig. S3), which is consistent with a recent report [26]. Freibe and colleagues [25],[72] reported that the insertion of CRE 5BSL3 at VR did not affect the replication of a sub-genomic replicon; however, deletion of VR region impaired genome replication. The poly(U/UC) tract has been reported critical for viral replication [25],[26],[27],[42],[71]. The insertions at 3′NTR could possibly affect the binding of cellular factors involved in RNA genome replication leading to attenuating or lethal phenotype.

### Validating the phenotypes identified by the functional profiling screen

**Figure 6:**
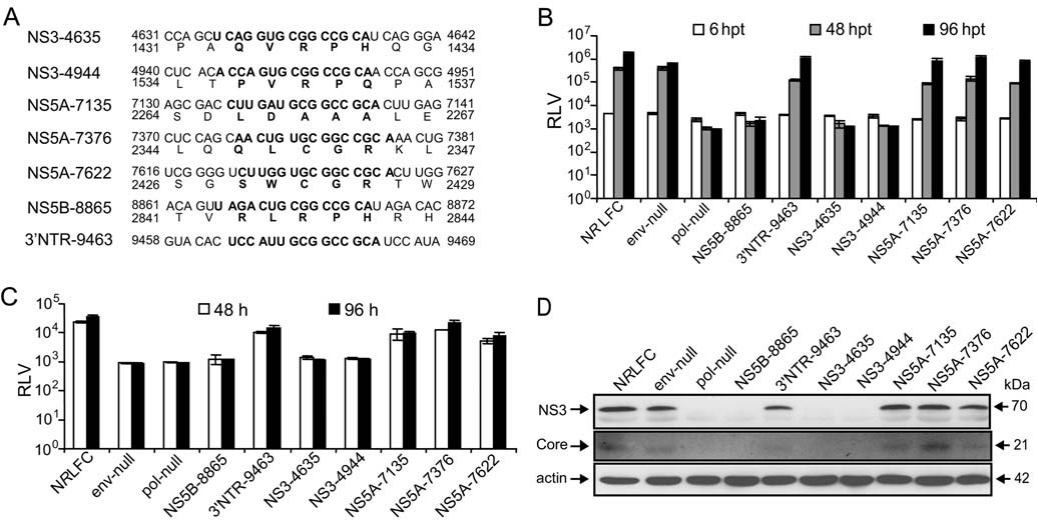
Validating the HCV functional profiling phenotypes by individual mutant viruses. (A) Nucleotide and amino acid sequence information for the 15-nt insertions engineered in the individual mutant N*R*LFC reporter viruses is shown. The inserted nucleotide/amino acid sequences are shown in bold face. The genomic position of nucleotide and amino acid residues are indicated. (B) Analysis of viral genome replication of mutant viruses. 10 µg of *in vitro* transcribed genomic RNA of wild-type N*R*LFC reporter virus and the mutant reporter viruses were individually introduced into Huh-7.5.1 cells by electroporation. The mutant reporter viruses lacking envelope (env-null) and polymerase activity (pol-null) are included as controls. The transfected cells were lysed at indicated time points using Promega passive lysis buffer, and the levels of *Renilla* luciferase were quantified. The experiment was done in triplicate and the mean values with standard deviation of *Renilla* luciferase values (RLV) are presented as a bar graph in log_10_ scale. (C) Measuring the production of infectious viral particles by mutant viruses. The cell-free supernatants harvested at 48 and 96 hours post-transfection (hpt) were inoculated onto naïve Huh-7.5.1 cells. At 48 hpt the cells were lysed and the *Renilla* luciferase activities were assayed. The mean RLV with standard deviations are shown in the graph. The replication deficient mutants show only background level of luciferase activity. (D) The expression of HCV non-structural and structural proteins. The protein lysates obtained at 96 hpt were subjected to western blotting. The HCV core and NS3 antigens were detected by primary mouse monoclonal antibodies and secondary goat-anti mouse IgG conjugated with HRP. β-actin was included as a loading control.

We focused on the NS3, NS5A, NS5B, and 3′NTR regions for validating the 15-nt insertion phenotypes because of their critical role in viral genome replication. The nucleotide/amino acid sequences of the mutations that were introduced in the HCV genome are shown in Fig. 6A. The functional profiling analysis showed that the 15-nt insertions at NS3-4635 and NS3-4944 (helicase domain) and NS5B-8865 (thumb domain) resulted in a lethal phenotype. The 15-nt insertions at NS5A region-2 (nt 7135) and region-3 (nt 7376, nt 7622) and 3′NTR (nt 9463) were tolerated for virus replication (Fig. S4). The effect of these mutations on virus replication was validated with individual mutant viruses based on a monocistronic *Renilla* luciferase HCV reporter virus, N*R*LFC (Fig. S8). The mutant reporter viruses defective in RNA polymerase activity (pol-null) and envelope (env-null) were included as controls for RNA genome replication and infectious virus production. The genome replication and the supernatant infectivity of mutant viruses were assessed by measuring the *Renilla* luciferase activity of the transfected and infected Huh-7.5.1 cells at the indicated time points, respectively (Fig. 6 B,C). The core and NS3 antigen expression of transfected cells was tested at 96 hpt (Fig. 6D). The mutant viruses demonstrated a consistent phenotype with that of the genome-scale functional profiling analysis, confirming the usefulness of this system. Similar results were also obtained with JFH-1 based mutants (data not shown). Our study using JFH-1 mutant viruses containing alanine-substitution of core C-terminal transmembrane domain showed that several residues were dispensable for infectious virus production, further validating our functional profiling system (RR and VA unpublished observation). As this experiment was completed, an independent study reported similar findings [13].

### Role of 3′NTR variable region for viral replication

**Figure 7:**
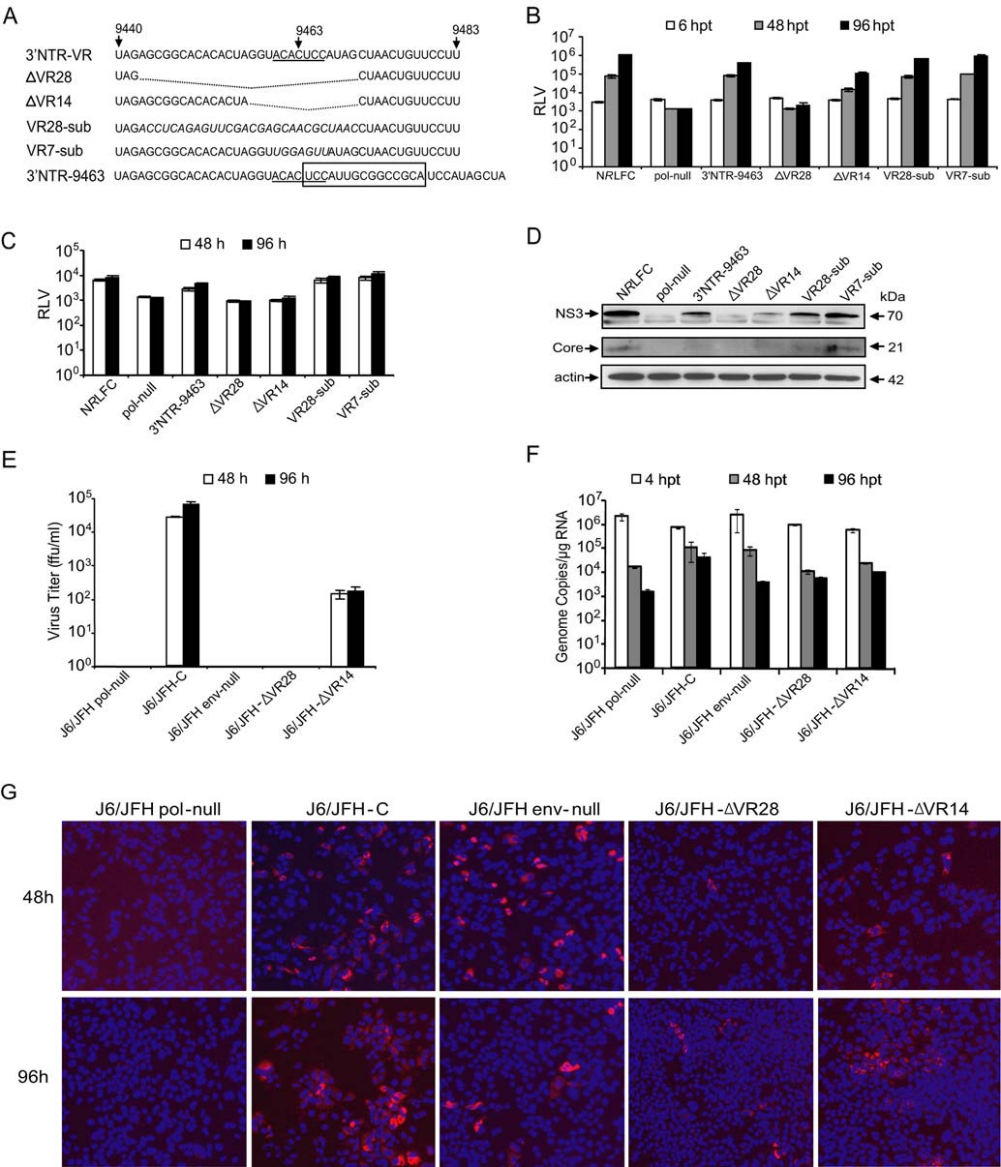
Analysis of 3′NTR Variable Region in HCV replication. (A) Nucleotide sequence of JFH-1 3′NTR variable region and the mutations engineered in the individual mutant viruses are depicted. The genome position of the nucleotides is indicated. The sequence that is conserved across all the genotypes is underlined. The deleted polynucleotide regions are shown in dotted lines. The substituted heterologous polynucleotides are in italics. 15-nt insertion sequence is boxed. Due to space limitation, only a partial sequence is shown for 3′NTR-9463 mutant. (B) Analysis of viral genome replication of mutant reporter viruses. The mean *Renilla* luciferase values (RLV) with standard deviations are shown in the graph (log_10_ scale). (C) Measuring the production of infectious viral particles by mutant reporter viruses. The mutants deficient in production of infectious particles show only a background level of luciferase activity. (D) Western blotting analysis of viral proteins, NS3 and core, expression. (E) Comparison of J6/JFH-C mutant viral infectivity. The virus titer (ffu/ml) of cell-free supernatant collected at 48 and 96 hpt of J6/JFH-C based mutants' transfected cell culture was measured by infecting naïve Huh-7.5.1 cells. Mean values and standard deviations are shown in the graph. (F) Comparison of J6/JFH-C mutant viral genome replication. At 4, 48, and 96 hpt total cellular RNAs were harvested and subjected to RT-qPCR. The genome copy numbers per µg of RNA are presented. (G) Immunofluorescence assay. At 48 and 96 hpt the cells were fixed and stained for HCV core antigen. The cell nuclei were visualized by DAPI staining. For B, C, and D experimental details see Figure 6 legend.

The functional profiling of the 3′NTR revealed that an equal number of insertions at the VR exhibited either tolerated or attenuated phenotypes. An earlier study in chimpanzees has shown that a mutant virus lacking 24 nucleotides at the 3′NTR VR is replication-competent *in vivo* [27]. Subsequent studies showed that a sub-genomic replicon lacking partial or complete VR was viable, but genome replication was impaired significantly [25],[42]. We have found many of the insertions at the 3′NTR VR resulted in attenuation of virus replication. Furthermore, based on the kinetics of disappearance of viral mutants having altered poly(U/UC) and variable regions, we hypothesized that the 3′NTR plays a critical role in infectious particle production. To test this hypothesis we have constructed mutant reporter viruses having deletions of 14 nucleotides [ΔVR14 (nt 9457–9470)] or 28 nucleotides [ΔVR28 (nt 9443–9470)] or substitution of the VR, which encompasses a seven-nucleotide conserved sequence (Fig. 7A). The substitution mutants behaved like parental N*R*LFC reporter virus, while the deletion mutants had impaired RNA genome replication and viral particle production (Fig. 7B–D). Because the N*R*LFC reporter virus had a reduced infectivity compared to that of parental J6/JFH-C virus (Fig. S8), we tested the VR deletion mutants with J6/JFH-C background (Fig. 7E–G). The deletion of 14 nucleotides at VR resulted in a significant reduction in viral replication and infectious particle production. Although the J6/JFH-ΔVR28 virus had a lower level of genome replication and core antigen expression (Fig. 7F,G), there was no detectable amount of infectious virus in the supernatant. Furthermore, the wild-type virus transfected cells underwent growth arrest and dead cells/cell debris were detected in the culture medium indicating CPE. The cell growth arrest and CPE were not observed in J6/JFH-ΔVR28 and J6/JFH-ΔVR14 mutant genome transfected cells. These results suggest that the 3′NTR VR mediated-spacing is important for efficient viral replication and infectious virus production. Taken altogether, the functional profiling system effectively identified viral functional domains throughout the HCV genome.

## Discussion

We have described a high-throughput, quantitative mutational analysis system to identify HCV protein domains and *cis*-acting elements that are essential or non-essential for virus replication in cell culture. We took advantage of advances in DNA analyzing technology to obtain the whole genome functional profile of HCV. We have characterized a library of mutants with 2399 random insertions between nt 55 and 9571 of HCV genome. Approximately 80% (1914), 4.6% (111), and 15.6% (374) of the insertions had lethal, attenuating, and tolerated phenotypes, respectively. Most of our data is consistent with previous studies using individual mutants, which validates our approach [8],[13],[25],[26],[27],[42],[43],[46],[54],[62]. The phenotypes identified through transposon-insertion and conventional site-specific (deletion and substitution) mutagenesis could differ in some instances due to the nature of genetic changes.

Among other members of *Flaviviridae*, while deletion of the 3′NTR variable region of dengue virus resulted in severe attenuation of virus growth [74], there was no effect of such deletion on tick-borne encephalitis virus replication [75]. We have dissected the role of 3′NTR VR during virus replication by insertion mutagenesis, deletions, and substitutions. Deletion of VR resulted in reduction in viral genome replication and infectious viral particle production. Based on the substitution study at the 3′NTR VR, the stem loop structure, if present, is not essential for genome replication and virion morphogenesis and release. A previous study has shown that the conserved seven nucleotides at the VR are dispensable for RNA replication [29]. We found that these conserved nucleotides do not have a direct role in both viral RNA genome replication and infectious virion production in cell culture. On one hand, the deletion of the 3′NTR VR results in the reduction of genome replication; on the other hand, replacement of the deleted 3′NTR VR region with heterologous polynucleotides results in restoration of genome replication to that of wild-type virus, indicating that irrespective of nucleotide sequence, the spacing between the region up- and downstream of 3′NTR VR region is important for efficient RNA replication. The VR might have a role in efficient genome packaging or binding of RNA replication machineries onto the 3′NTR during virus replication in the host cell.

We have identified several novel regions in 5′NTR, p7, NS3, NS4B, NS5A, NS5B, and 3′NTR that tolerate the insertions for virus replication in cell culture. These domains, however, could play important roles in *in vivo* infection, including immune evasion. Defining regions critical and less-critical for viral replication at the genome scale will facilitate the rational design of vaccine candidates. The HCV regions with attenuating insertions could be further characterized by deletion mapping. Attenuating deletions along with mutations that inactivate immune evasion domains can be combined into developing a live virus vaccine that is attenuated and non-recombinogenic.

In order to complete the viral lifecycle, the virus hijacks and/or counteracts cellular functions, including signaling pathways, cell cycle regulations, and innate and adaptive immune responses. Our approach can be used to define such interactions. For example, comparing mutational profiles of HCV mutant libraries selected in cells that are deficient in an innate immune factor would facilitate identifying viral determinant(s) that counteract or modulate the host response pathway. The *in vivo* role of tolerated insertions could be dissected by passaging and profiling the HCV mutant library in the human liver cell-grafted mouse model or a primate model. Moreover, modeling the HCV protein structures based on mutational profiles could be useful to elucidate the structure-function relationship of individual HCV proteins. These future investigations would shed light on the mechanism of HCV replication. Furthermore, the high-throughput mutational analysis platform would be a useful tool for the functional genomics study of other RNA and DNA viruses.

## Materials and Methods

### Cells

The Huh-7.5.1 cell line (a kind gift from Dr. Francis Chisari, The Scripps Research Institute, La Jolla) was cultured in complete DMEM containing 10% fetal bovine serum, 10 mM non-essential amino acids (Invitrogen, Carlsbad, USA), 10 mM Hepes, penicillin (100 units/ml), streptomycin (100 mg/ml), and 2 mM L-glutamine at 37°C with 5% CO_2_.

### Virus and Plasmid Constructs

The plasmid containing the complete genome of a HCV GT2a strain JFH-1 (kindly provided by Dr. Takaji Wakita, National Institute of Infectious Diseases, Japan), was used for construction of recombinant viruses. An intra-genotype chimeric virus, pJ6/JFH-C, comprising 5′NTR, structural regions and part of non-structural regions (p7 and partial NS2) of the J6CF strain (NCBI accession no. AF177036) and non-structural regions of JFH-1 strain was generated. The J6CF genomic region, nt 1 to 2878, was synthesized by PCR based assembly of oligonucleotides (Invitrogen). The T7 promoter sequence (5′-TAATACGACTCACTATAG-3′) and nt 2879 to 2967 of the JFH-1 isolate was engineered at the 5′ and 3′ ends of the J6CF 1-2878 fragment, respectively. The final assembled PCR product (T7-J6CF/JFH) was cloned into pZero-blunt vector (Invitrogen) and sequence verified. The *Eco*RI- *Not*I fragment (2.9 kb) of the pJFH-1 was swapped with the assembled T7-J6CF/JFH fragment to obtain pJ6/JFH-C. A monocistronic chimeric reporter virus, pN*R*LFC, which was based on pJ6/JFH-C parental virus, was constructed. A plasmid containing a reporter cassette, T7-5′NTR (388 nucleotides)-*Renilla* luciferase gene-F2A seqeuce-Core-E1, was constructed. The F2A sequence introduced was 5′-GTGAAACAGACTTTGAATTTTGACCTTCTCAAGTTGGCCGGAGACGTCGAGTCCAACCCTGGGCCC -3′. The *Eco*RI-*Bsi*WI fragment containing the reporter cassete was subcloned into pJ6/JFH-C to construct pN*R*LFC. To construct the envelope-null mutant virus, an in-frame deletion of nt 1040–2215 was engineered in the pJ6/JFH-C genome. This deletion removed most of the E1 and E2 coding regions. An identical E1 and E2 deletion mutant reporter virus pN*R*LFC was also constructed. An RNA polymerase-null virus with pJ6/JFH-C or pN*R*LFC background was constructed by mutating the catalytic residues GDD to AAG amino acid residues. The *Not*I restriction enzyme site present in JFH1 genome at nt 2955 was abolished by PCR-mediated introduction of silent point mutations ( CGGC-> TGGT ). These point mutations did not affect the virus infectivity (data not shown). This plasmid was subjected to *in vitro* Mu-transposon mediated mutagenesis (MGS kit, Finnzymes). The location of transposon insertion in the JFH-1 genome was identified by sequencing using a transposon-specific primer (5′-CAGAGATTTTGAGACACAACGT-3′). The plasmids having a transposon insertion at NS3-4635, NS3-4937, NS5B-8865, NS5A-7622, and 3′NTR-9463 were included for *Not*I restriction digestion to remove the transposon fragment, resulting in only 15-nt remaining at the insertion site. PCR-mediated site-specific mutagenesis was employed to introduce several mutations into the viral genome. The mutant JFH-1 plasmids containing a 15-nt insertion at pJFH-1 NS5A regions, NS5A-7135 (5′-CTTGATGCGGCCGCA-3′) and NS5A-7376 (5′-AACUGUGCGGCCGCA-3′) were constructed. The pJFH-1 mutant plasmid with substitution of 7 nucleotides (nt 9460–9467) or 28 nucleotides (nt 9443–9470) at 3′NTR were constructed. The pJ6/JFH-C based mutant plasmids having deletion of 14 nucleotides [(nt 9457–9470) pJ6/JFH-ΔVR14] or 28 nucleotides [(nt 9443–9470) pJ6/JFH-ΔVR28] at 3′NTR were constructed. To construct reporter mutant viruses, the *EcoR*I −*Avr*II fragment of the pJFH-1 and pJ6/JFH-C based mutant plasmids was replaced with that of the reporter virus pN*R*LFC. The sequence information of the primers used for the construction of mutant viruses will be available upon request.

### *In vitro* Transcription and RNA Transfection

The viral plasmids linearized by *Xba*I restriction enzyme and treated with mung bean nuclease (New England Biolabs, Beverly, USA) were subjected to *in vitro* transcription using T7 Ribomax Express Large Scale RNA Production System according to the manufacturer's instructions (Promega Corporation, Madison, USA). A total of 16 µg of the pJFH-1 insertion library DNA was used for *in vitro* transcription. The DNase-treated RNA were purified and stored at −80°C in aliquots. The *in vitro* transcribed RNAs were electroporated into Huh-7.5.1 cells. Briefly, the Huh-7.5.1 cells were trypsinized and washed twice with ice cold Opti-MEM transfection media (Invitrogen) and resuspended in Opti-MEM at 1×10^7^ cells per ml. 10 µg of *in vitro* transcribed RNA was mixed with 400 µl of cells in 0.4 cm electroporation cuvettes. Electroporation was conducted by using a BioRad elecroporator with the settings of 270 V, 100 ohms, and 960 µF. Subsequently, the cells were resuspended in 40 ml of complete DMEM and plated in T-75 flasks and 48-well plates. At 8 hpt, media containing dead cell debris in the culture flasks and plates were replaced with fresh complete DMEM.

### Generation of 15-nt Insertion JFH-1 Plasmid Library

The plasmid pJFH-1lacking *Not*I site, was subjected to *in vitro* Mu-transposon mediated mutagenesis (MGS kit, Finnzymes). A total of 4.7×10^5^ individual bacterial colonies were obtained and the mutant plasmids were isolated from the pooled bacterial colonies. To remove the transposon DNA fragment, 5 µg of the pooled mutant plasmids were subjected to *Not*I digestion, self-ligation, and selection in bacteria. This resulted in a library of mutants having a 15-nt sequence, 5′-NNNNNTGCGGCCGCA-3′ (N: duplicated 5 nucleotides from target DNA), inserted randomly in the pJFH-1 plasmid.

### Genetic Selection of Mutant JFH-1 Library in cell culture

A total of 16 µg of the pJFH-1 library DNA was used for *in vitro* transcription. 120 µg of DNase-treated RNA was delivered into 4.8×10^7^ Huh-7.5.1 cells by electroporation. The cells were plated in twelve T-150 flasks and were split into forty T-150 flasks at 1∶3 or 1∶4 ratios on 4, 7, 10, 13, and 16 dpt. During each split, ∼one third of the pooled cells was saved for RNA isolation. The selection was terminated at 21 dpt, when many of the cells were started showing CPE [34],[41]. Total RNA was isolated from the cells using Tri-reagent (Molecular Research Center Inc. Cincinnati, OH). The DNase-treated and -purified RNA was used for functional profiling analysis.

### Functional Profiling Analysis of the HCV genome

A total of 65 µg of RNA from each of the non-selected and cell culture selected JFH-1 mutant RNAs were reverse transcribed by Superscript III Reverse Transcriptase (Invitrogen) using random hexamers. The cDNAs and pJFH-1 library DNA (included to ascertain the library complexity) were used as templates for PCR amplification of thirteen overlapping fragments using JFH-1 specific primers (Table S1). Each fragment has an overlap of ∼200 nt with flanking fragments. Fifty nanograms of purified RT-PCR or PCR product was used as a template for a second PCR with an insertion-specific mini-primer (5′-TGCGGCCGCA -3′), which has 5′ end labeled with a fluorescent dye-VIC (Applied Biosystems), and one of the JFH-1 fragment specific primers (Table S2). A total of forty-eight JFH-1 specific primers, designed at approximately 200 nt intervals, were used. Each of the JFH-specific primer and mini-primer combinations tested negative for generating any spurious PCR products using wild-type JFH-1 genome template. For each primer, the PCR reactions were done in duplicate. The conditions used for the second PCR were 95°C for 5 min (1 cycle); 95°C for 1 min, 52°C for 1 min and 72°C for 2 min (35 cycles); 72°C for 20 min (1 cycle). The fluorescent-labeled PCR products were analyzed in duplicate with Liz-500 size standard (Applied Biosystems) by using a 96-capillary genotyper (3730xl DNA Analyzer, Applied Biosystems) at the UCLA Genotyping and Sequencing core facility.

### Data Processing and Interpretation

The data generated by the capillary genotyper were processed by GeneMapper software (Applied Biosystems) using Amplified Fragment Length Polymorphism analysis tool. The normalized data was visualized by an electropherogram and exported as a data file. For each PCR sample, the exported data contained information regarding the PCR product size at nucleotide level and peak area. The exact position of an insertion in the genome, for each of the 48 gene specific primer-generated PCR products, was calculated by subtracting 15-nt from the size of the particular PCR product and adding the HCV genome position of the specific primer. Comparison of the 15-nt insertion sites identified in the mutated HCV genome by PCR profiling and sequencing revealed that the accuracy of the PCR profiling is within one to two nucleotide(s). For each sample, the PCR profiles were consistent among duplicates and representative data were used for obtaining final assembly. To assemble the locations of insertion sites for the entire HCV genome, the insertion profiles obtained between 50 to ∼250 nt for each specific primer was taken into account. To assign a phenotype for each insertion mutant, the ratio of peak area between selected (21 dpt) and non-selected pools was calculated. The incorporation of functional profile data into the crystal structure of HCV proteins and the generation of graphics were done using PyMOL Viewer program (DeLano Scientific, USA).

### Quantitative Reverse Transcription-PCR

A two-step reverse transcription-PCR (RT-PCR) was carried out for determining the HCV RNA copy number. Briefly, 1 µg of total cellular RNA was reverse transcribed by using Superscript III Reverse Transcriptase (Invitrogen) and HCV 5′UTR specific primers (JFH RTQ F: 5′- CTGGGTCCTTTCTTGGATAA-3′ and JFH RTQ R: 5′- CCTATCAGGCAGTACCACA-3′) as per the manufacturer's instructions. 100 ng of resulting cDNA was used as a template for the subsequent quantitative-PCR (Q-PCR) using QuantiTect Probe FAM 5′-GAGTAGCGTTGGGTTG-3′ (Qiagen). 10^1^ to 10^7^ copies of *in vitro* transcribed JFH-1 genomic RNA were reverse transcribed along with the samples, and were included as a standard for copy number determination during Q-PCR. The reaction was run at 95°C for 15 min (1 cycle), 95°C for 30 s, 55°C for 30 s, and 72°C for 10 s (45 cycles). The results were analyzed in Opticon II (MJ Research, Cambridge, MA).

### Measuring virus titer

The virus titer was measured by calculating the foci forming unit (ffu) of infectious viral particles per ml of cell-free culture supernatant. The infected-culture supernatant was 10-fold serially diluted in complete DMEM and inoculated in triplicate onto naïve Huh-7.5.1 cells (3×10^3^ cells/well) in 96-well plates. At 72 hours post infection (hpi), the cells were fixed and immunostained for HCV core antigen. The number of core antigen positive foci were counted at the highest dilution, and average foci forming units per ml was calculated.

### *Renilla* Luciferase Reporter Assay for Viral Genome Replication and Infectivity

For viral genome replication assay, the HCV RNA transfected cells were plated in triplicate in 48-well plates. The cells were lysed with passive lysis buffer (Promega) at 6, 48, and 96 hpt. The culture plates were gently rocked at room temperature for 15 min, then stored at −80°C. To determine the supernatant infectivity, 500 µl of cell-free supernatant obtained from HCV RNA transfected cells at 48 and 96 hpt was inoculated in triplicate onto naïve Huh-7.5.1 cells in 48-well plates. At 48 hpi the cells were lysed and stored at −80°C. 10 µl of lysate was used for measuring the *Renilla* luciferase activity using a *Renilla* Luciferase Assay System kit (Promega).

### Western blotting

For western blotting, the cell lysates were resolved by SDS-PAGE and transferred to a nitrocellulose membrane. The membranes were blocked (5% skim milk, 0.2% Tween-20 in PBS) and probed with mouse monoclonal antibody to core [(C7-50) Abcam], NS3 [(H23) Abcam)] and beta-actin (Sigma). Goat anti-mouse IgG conjugated with horseradish peroxide (Amersham Pharmacia Biotech) secondary antibody was detected by chemiluminescence (ECL Plus, Amersham Pharmacia Biotech).

### Immunofluorescence assay

The HCV infected or transfected cells were fixed with 4% paraformaldehyde. Following three PBS washes, the cells were blocked (3% goat serum, 3% BSA, 0.1% Triton-x 100 in PBS) and incubated with mouse monoclonal anti-core primary antibody (C7-50 (Abcam, Cambridge, USA)) at a dilution of 1∶300 for 5 hrs at 4°C. The goat anti-mouse IgG polyclonal antibody conjugated to Cy-3 was added as a secondary antibody (Jackson ImmunoResearch Laboratories, USA) at 1∶200 dilution and incubated for 1 hr at room temperature. Between antibody changes, the cells were washed thrice with PBS. The nucleus was stained with DAPI (Sigma).

## Supporting Information

Figure S1 The amino acid sequences encoded by 15-nt insertions at three possible reading frames. The duplicated nucleotides at the target site of the mini Mu-transposon insertions are underlined. The inserted sequences are shown in bold face. The amino acid (aa) sequences are in italics. The numbers corresponds to the amino acid positions of the JFH-1 genome. Note that the insertions do not introduce STOP codons in all of the reading frames.
(0.66 MB EPS)

Figure S2 Kinetics of JFH-1 library replication in Huh-7.5.1 cells. The HCV genome copy numbers and the virus titer [foci forming unit per milliliter (ffu/ml)] for the indicated time points are shown in the line and bar graphs, respectively. Mean values with standard deviations are presented (log_10_ scale).
(0.83 MB EPS)

Figure S3 Electropherogram depicting the effect of 15-nt insertions in NS5B-3′NTR of HCV. The X-axis shows the 15-nt insertion sites as corresponding peaks and the Y-axis shows the fluorescent signal intensity of the peaks. Nucleotide positions of the JFH-1 genome are numbered on the top. Schematic representations of the NS5B Transmembrane Domain (TMD) coding region, 3′NTR Variable Region (VR), and poly(U/UC) tract locations are depicted. The cDNA generated from the *in vitro* transcribed mutant RNA genomic library (RNA input) and JFH-1 mutant viral library selected in Huh-7.5.1 cell culture (selection 2, 4, 10, 16 and 21 dpt) were subjected to the functional profiling analysis. Comparison of electropherogram panels shows that all of the insertions at poly(U/UC) tract were negatively selected by 2 dpt. Insertions at the VR show a gradual reduction in replication fitness. Insertions at NS5B-TMD show positive or negative selection depending on the insertion site.
(0.03 MB PDF)

Figure S4 Genome scale functional profile of HCV. Graphical representation of location and phenotype of 15-nt insertions in the HCV genome are shown. The nucleotide and amino acid (in parenthesis) numbers correspond to the JFH-1 genome sequence. A schematic diagram of the HCV region is shown for each graph. For each 15-nt insertion mutant, the ratio of the peak area was calculated between selected (21 dpt) and non-selected pools and plotted in a bar graph as fold change (log_10_ scale). The lethal phenotype (critical region, red bar) is an absence of an insertion mutant in the selected population. The attenuated phenotype (less critical region, blue bar) denotes an over two-fold reduction in replication. The tolerated phenotype (dispensable region, green bar) is replication competent.
(0.12 MB PDF)

Figure S5 The predicted secondary structures of 15-nt insertions at 5′NTR domain IV. The tolerated insertions maintain an open confirmation similar to that of the wild-type domain IV, whereas lethal insertions form a stable stem loop structure. The asterisk indicates an insertion mutant not present in our screen. Insertions at nt-336 and nt-338 resulted in duplication of the AUG start codon.
(0.03 MB PDF)

Figure S6 The crystal structure of HCV proteins displaying functional profiling phenotypes. The amino acid residues were color coded for insertion phenotypes: red (lethal), blue (attenuating), green (tolerated), and grey (no insertion). (A) The ribbon diagrams depict a dimer of NS2 protease domain (amino acid residues 94-217) (PDB accession code 2hd0) [20]. (B) Ribbon and surface diagrams of HCV genotype 1 NS3 monomer are shown (PDB accession code 1CU1) [49]. The protease and helicase domains are indicated. (C) The genotype 1b, Con1 isolate NS5A domain 1 (PDB accession code 1ZH1) [56] ribbon and surface diagrams are shown (bottom, front and top views). The zinc atoms in NS3 and NS5A structures are colored in magenta. The subdomain(s) coordinating the zinc atom had tolerated insertions in both NS3 and NS5A proteins. The structure analysis and graphics generation were done using PyMOL Viewer.
(5.03 MB EPS)

Figure S7 The 15-nt insertions tolerated for HCV replication at NS4B/5A and NS5A/5B cleavage sites. The nucleotide and the predicted amino acid sequences are shown. The number indicates JFH-1 genome position. The insertion sequences are bold faced. Insertions tolerated at the NS4B/5A (A) and NS5A/5B (B) cleavage sites are shown. The cleavage site is indicated by an arrow. Note that the insertions do not disrupt the critical P1-P1′ cleavage residues Cys-Ser (C-S). Asterisks indicate the insertion mutants that were not present in our screen. The function of amino acid residues at the N- and/or C-terminal of many HCV proteins was not affected by the insertions. The 15-nt insertion does not introduce a stop codon for any of the three reading frames: eg., insertions at nucleotides 6262, 6263 and 6264.
(0.40 MB TIF)

Figure S8 Analysis of genotype 2a chimeric parental and mono-cistronic *Renilla* Luciferase reporter Hepatitis C viruses. (A) Schematic representation of Hepatitis C viruses used in this study. The HCV non-coding and coding regions are depicted. The J6/JFH-C virus contains 5′ nontranslated region (NTR), structural, p7, and partial NS2 regions from J6CF strain of GT 2a HCV (dark grey), and non-structural region from JFH-1 strain of GT 2a HCV (mild grey). A mono-cistronic *Renilla* luciferase (*R*Luc) reporter virus (N*R*LFC) based on J6/JFH-C virus is shown. The *Renilla* luciferase gene is fused in frame with the core region through Foot and Mouth Disease virus 2A sequence (F2A). The mutant J6/JFH-C and reporter viruses, with envelope coding regions (E1 and E2) deleted and NS5B polymerase catalytic amino acid residues GDD (Gly-Asp-Asp) mutated to AAG (Ala-Ala-Gly) residues are shown. (B) Comparison of viral genome replication. The *in vitro* transcribed J6/JFH-C virus and the NRLFC reporter virus genomic RNAs were introduced into Huh-7.5.1 cells by electroporation. At 4, 48, and 96 hpt total cellular RNAs were harvested and subjected to RT-qPCR using HCV specific QuantiTect probe. The genome copy numbers were calculated per µg of RNA and presented as a graph. (C) Comparison of viral infectivity. The virus titer (ffu/ml) of cell-free supernatant collected at 48 and 96 hpt was measured by infecting naïve Huh-7.5.1 cells. Compared to the parental J6/JFH-C virus, the reporter virus had similar levels of genomic RNA replication, but was 10–100 fold attenuated in infectious particle production. C, core; E, envelope; NS, non-structural.
(2.89 MB EPS)

Table S1 Primers and the location of HCV fragments
(0.02 MB PDF)

Table S2 Primers used for Functional Profiling analysis of HCV genome
(0.03 MB PDF)
