# Supplementary figures and images for: High-Resolution Functional Profiling of Hepatitis C Virus Genome
Source: PLoS Pathog. 2008 Oct 17;4(10):e1000182. doi: 10.1371/journal.ppat.1000182 (PMC2564836; doi:10.1371/journal.ppat.1000182)

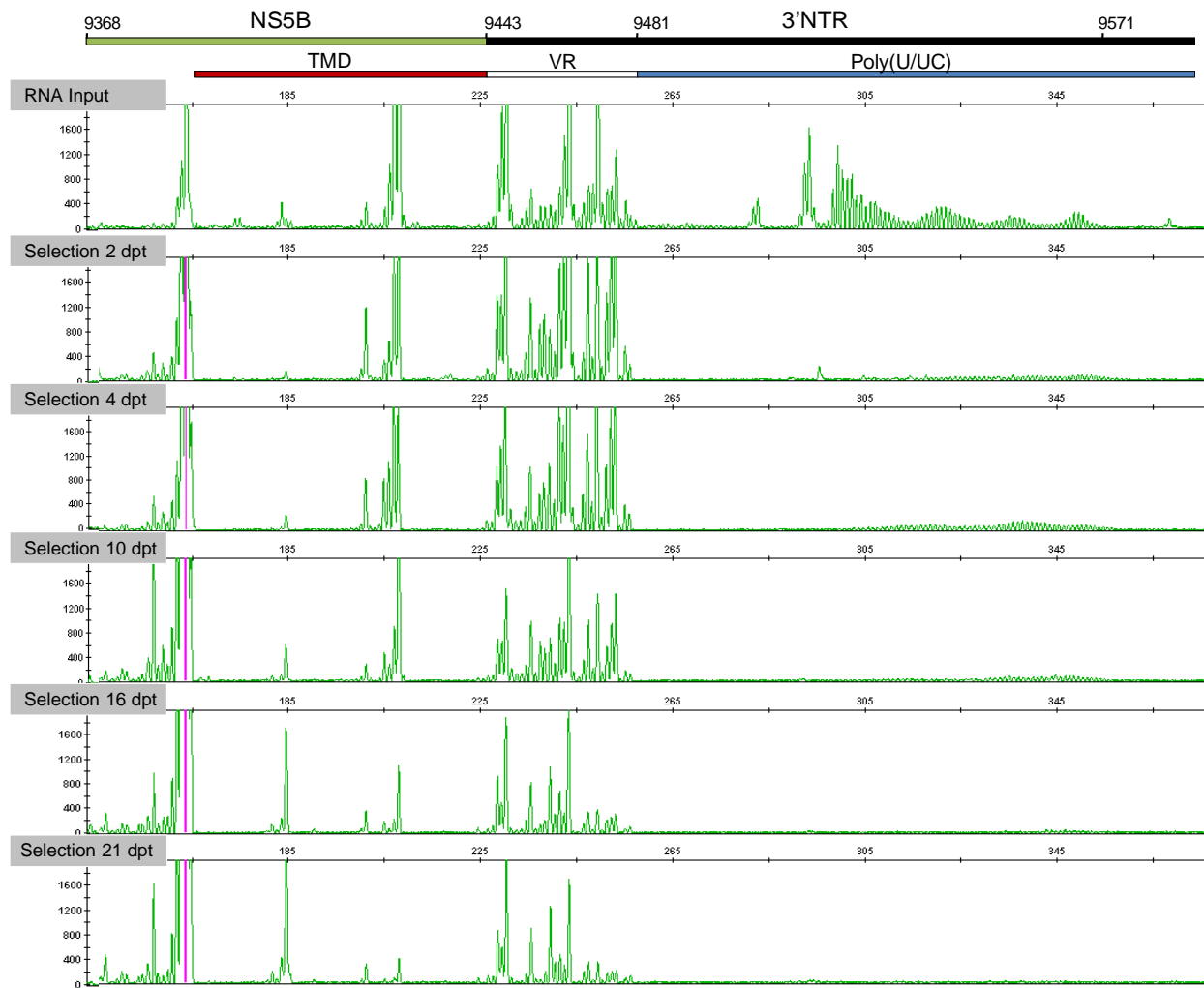

Supplement: Figure S3 — Electropherogram depicting the effect of 15-nt insertions in NS5B-3′NTR of HCV. The X-axis shows the 15-nt insertion sites as corresponding peaks and the Y-axis shows the fluorescent signal intensity of the peaks. Nucleotide positions of the JFH-1 genome are numbered on the top. Schematic representations of the NS5B Transmembrane Domain (TMD) coding region, 3′NTR Variable Region (VR), and poly(U/UC) tract locations are depicted. The cDNA generated from the in vitro transcribed mutant RNA genomic library (RNA input) and JFH-1 mutant viral library selected in Huh-7.5.1 cell culture (selection 2, 4, 10, 16 and 21 dpt) were subjected to the functional profiling analysis. Comparison of electropherogram panels shows that all of the insertions at poly(U/UC) tract were negatively selected by 2 dpt. Insertions at the VR show a gradual reduction in replication fitness. Insertions at NS5B-TMD show positive or negative selection depending on the insertion site. (0.03 MB PDF) [file ppat.1000182.s003.pdf]

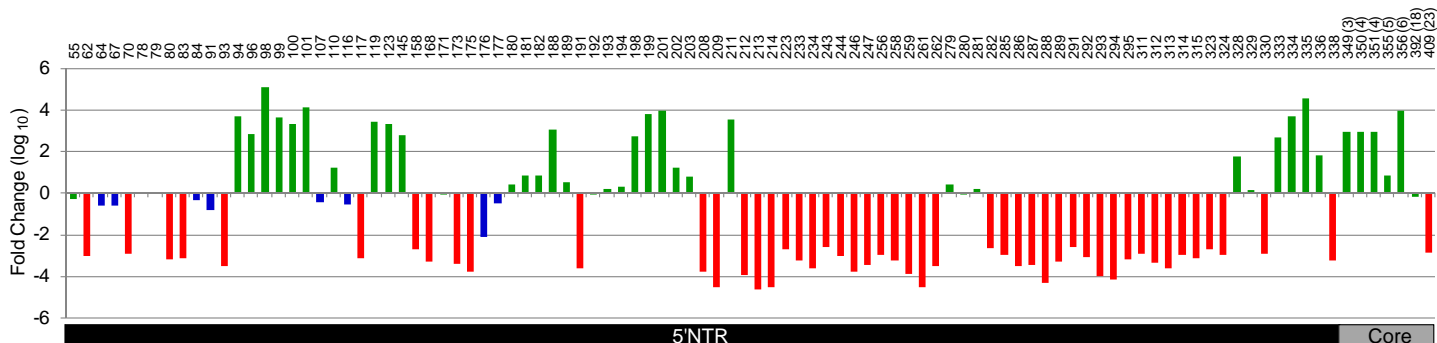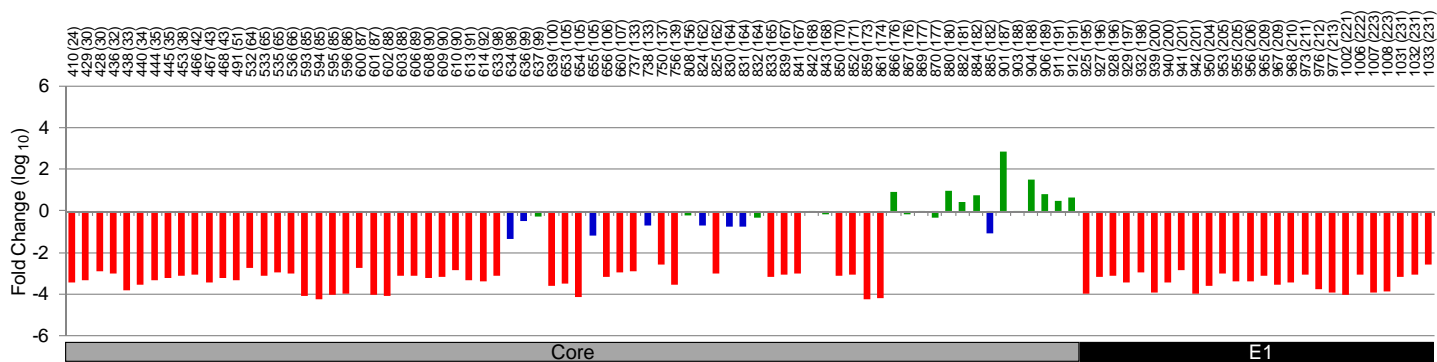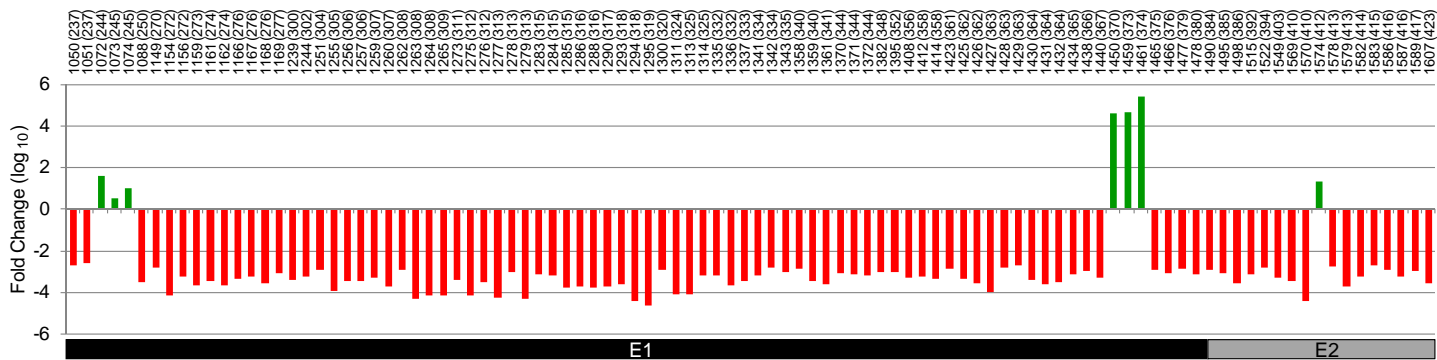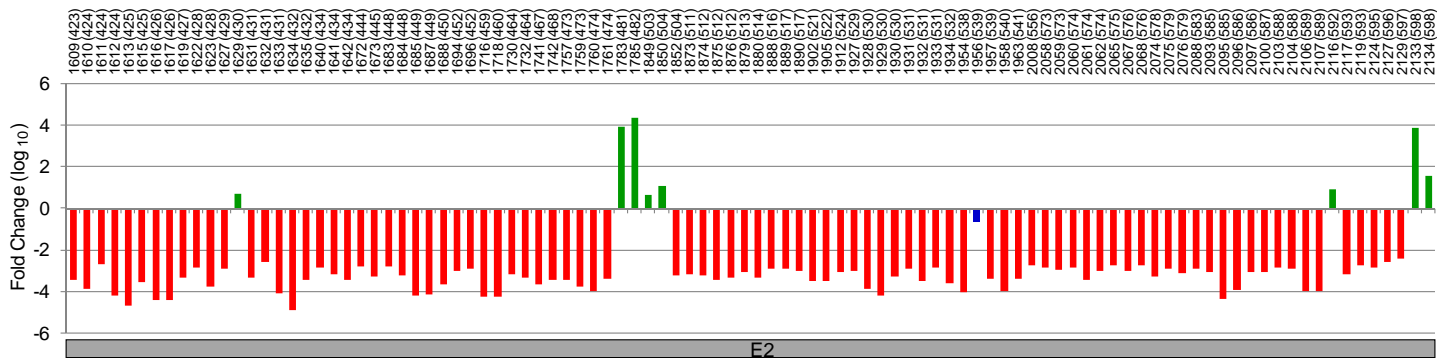

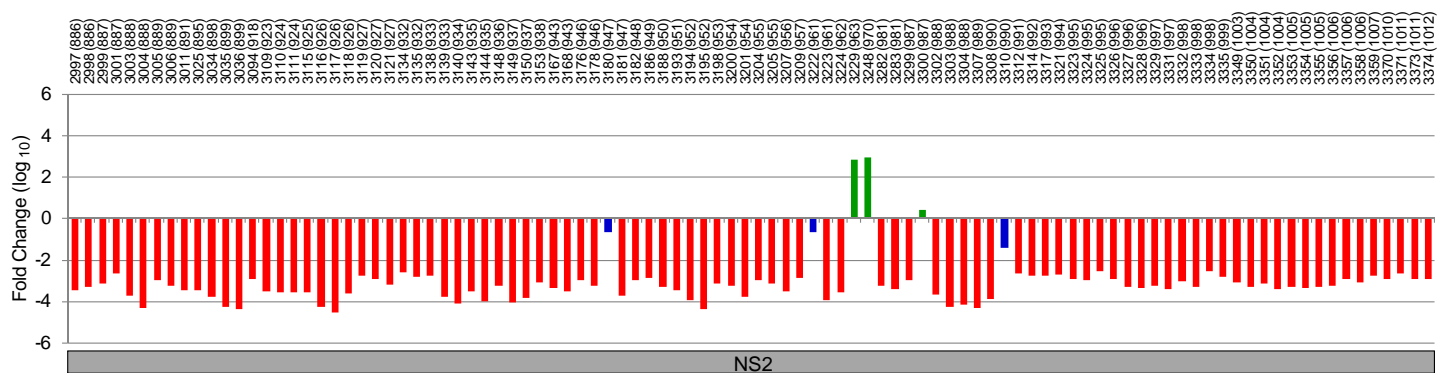



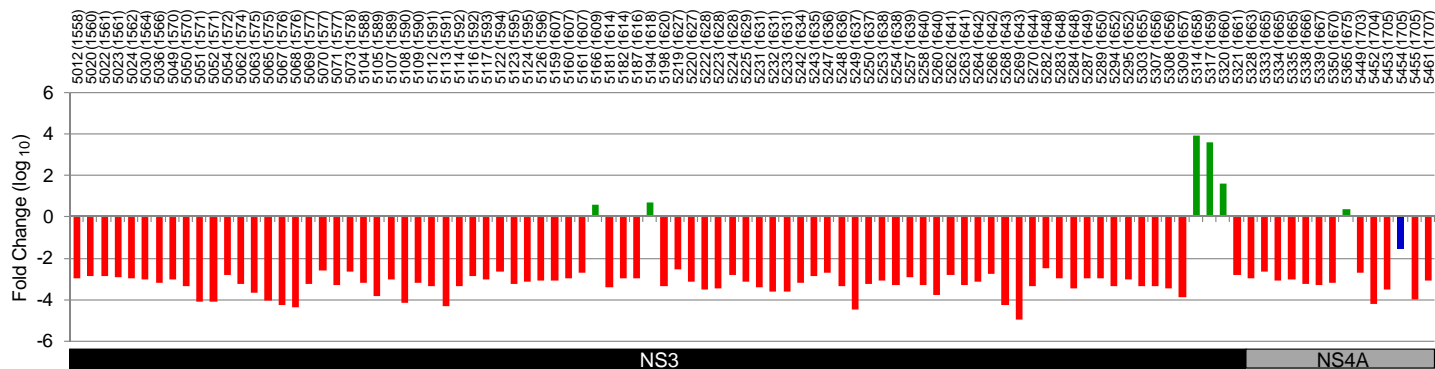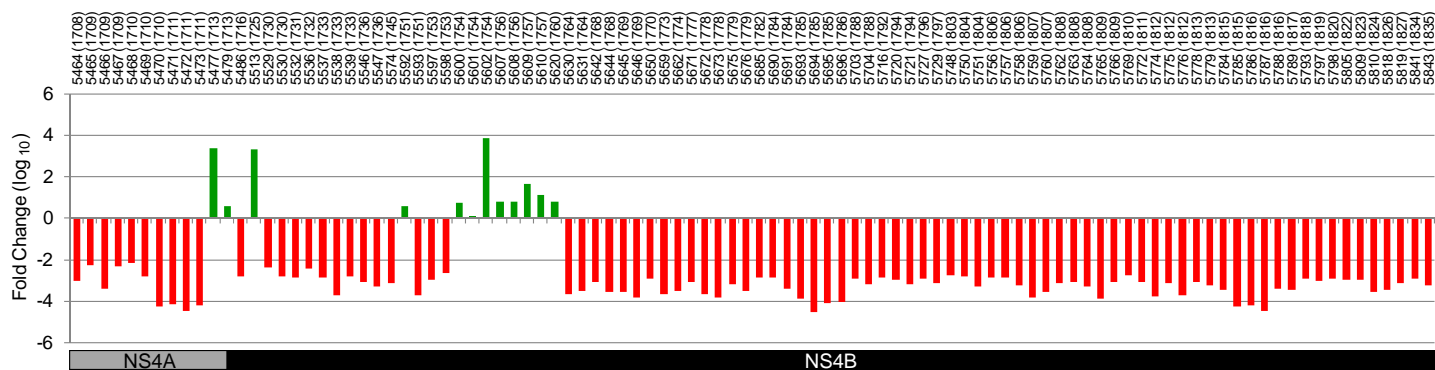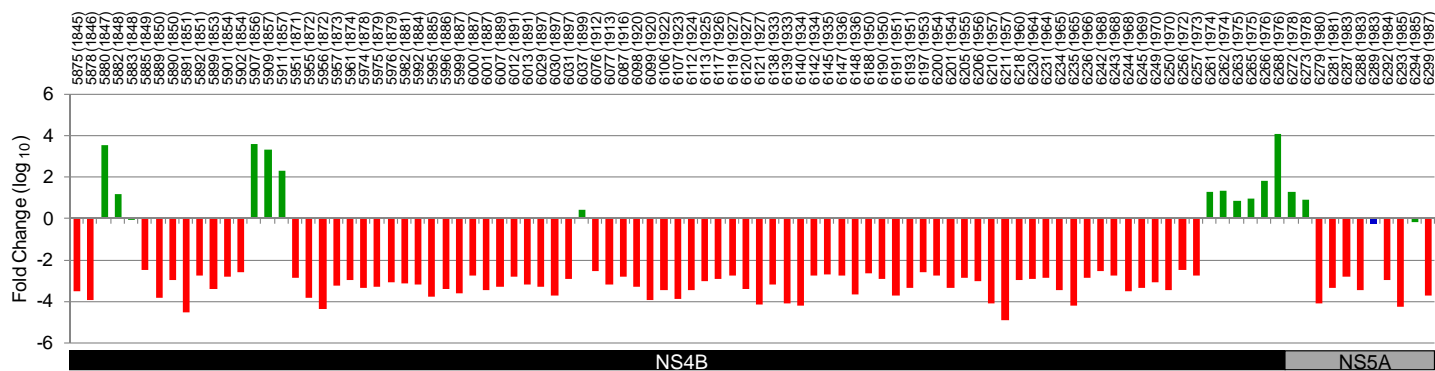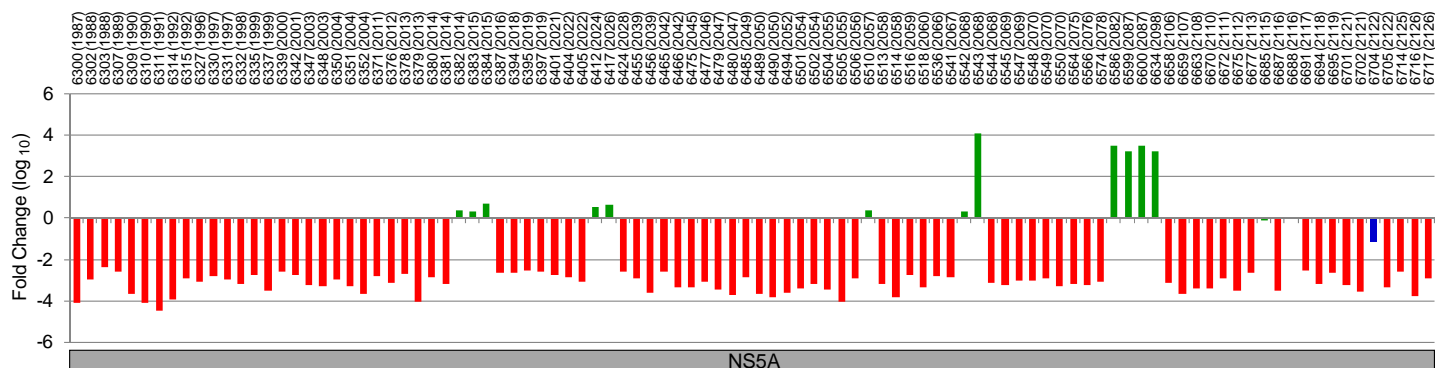

Supplement: Figure S4 — Genome scale functional profile of HCV. Graphical representation of location and phenotype of 15-nt insertions in the HCV genome are shown. The nucleotide and amino acid (in parenthesis) numbers correspond to the JFH-1 genome sequence. A schematic diagram of the HCV region is shown for each graph. For each 15-nt insertion mutant, the ratio of the peak area was calculated between selected (21 dpt) and non-selected pools and plotted in a bar graph as fold change (log10 scale). The lethal phenotype (critical region, red bar) is an absence of an insertion mutant in the selected population. The attenuated phenotype (less critical region, blue bar) denotes an over two-fold reduction in replication. The tolerated phenotype (dispensable region, green bar) is replication competent. (0.12 MB PDF) [file ppat.1000182.s004.pdf]

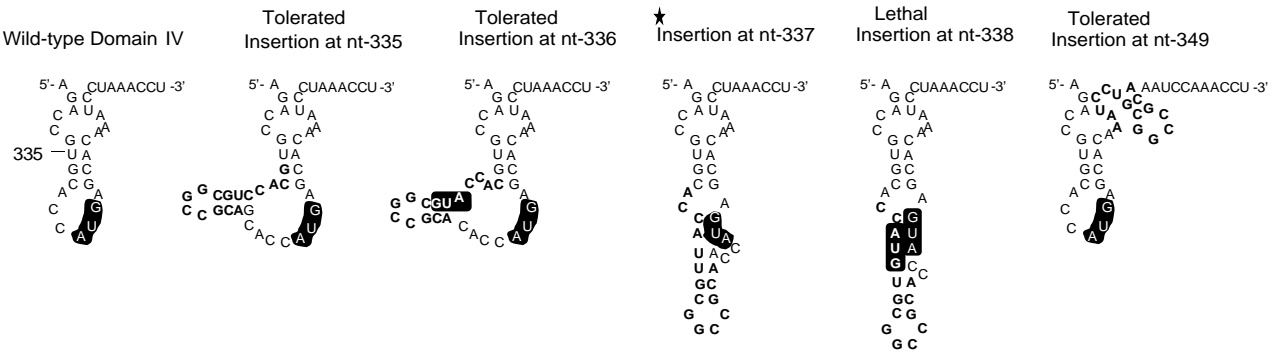

Supplement: Figure S5 — The predicted secondary structures of 15-nt insertions at 5′NTR domain IV. The tolerated insertions maintain an open confirmation similar to that of the wild-type domain IV, whereas lethal insertions form a stable stem loop structure. The asterisk indicates an insertion mutant not present in our screen. Insertions at nt-336 and nt-338 resulted in duplication of the AUG start codon. (0.03 MB PDF) [file ppat.1000182.s005.pdf]

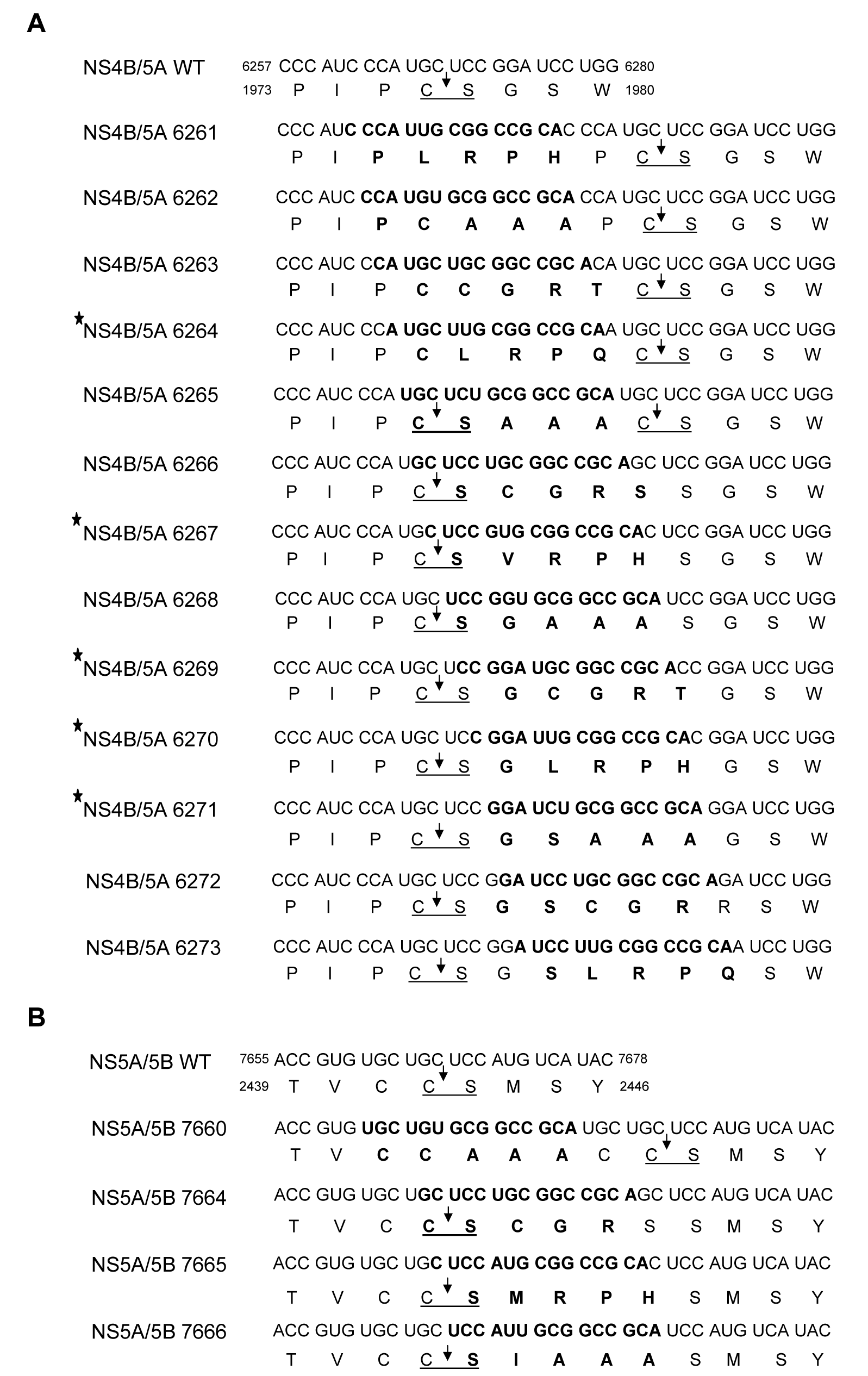

Supplement: Figure S7 — The 15-nt insertions tolerated for HCV replication at NS4B/5A and NS5A/5B cleavage sites. The nucleotide and the predicted amino acid sequences are shown. The number indicates JFH-1 genome position. The insertion sequences are bold faced. Insertions tolerated at the NS4B/5A (A) and NS5A/5B (B) cleavage sites are shown. The cleavage site is indicated by an arrow. Note that the insertions do not disrupt the critical P1-P1′ cleavage residues Cys-Ser (C-S). Asterisks indicate the insertion mutants that were not present in our screen. The function of amino acid residues at the N- and/or C-terminal of many HCV proteins was not affected by the insertions. The 15-nt insertion does not introduce a stop codon for any of the three reading frames: eg., insertions at nucleotides 6262, 6263 and 6264. (0.40 MB TIF) [file ppat.1000182.s007.tif]
