# Supplementary material for: High-Resolution Functional Profiling of Hepatitis C Virus Genome
Source: PLoS Pathog. 2008 Oct 17;4(10):e1000182. doi: 10.1371/journal.ppat.1000182 (PMC2564836; doi:10.1371/journal.ppat.1000182)
Supplement: Table S1 — Primers and the location of HCV fragments (0.02 MB PDF) [file ppat.1000182.s009.pdf]

Table S1. Primers and the location of HCV fragments

Fragment	Regions covered	Genome location	Amplicon size (nt)	Forward Primer (5'-3')	Reverse Primer (5'-3')
F1	5'NTR-C-E1	20-1059	1040	GACACTCCGCCATGAATCACT	ACCCAACACCGTGACGTATT
F2	E1-E2	815-1905	1091	GAGGACGGGGTTAATTATGCAA	TCGGTCGTGCCCACTACTA
F3	E2-p7	1672-2724	1053	CAACCGCTTTAACTCGTCA	GTGAGGCAATAGGTGGTCAA
F4	p7-NS2	2464-3497	1034	CCTCTCACCTGCTATCACAA	CCGTCATACTCACCACTATG
F5	NS2-NS3	3263-4301	1039	GTGGAACCCATCATCTTCA	CATCGCATATGATGATGTCATAG
F6	NS3	4030-5063	1034	GTACTTGCATGCTCCAACT	GGGCGTCTATGTGTGTGA
F7	NS3-NS4B	4822-5890	1069	CACAGGTAGAGGAAGACA	CTTACCCAGGCCTATGCTG
F8	NS4B-NS5A	5615-6682	1068	CCCAAAGTGGAACAATTTTG	CAGATTGTCAGTGGTCAGTC
F9	NS5A	6450-7507	1058	CCAACATCTCTGGCAATGT	GGCGGAACCTGTCTCTGA
F10	NS5A-NS5B	7229-8251	1023	CTCGTGGAATCGTGGAGGA	GTACTGGAAGCCATAGGAAG
F11	NS5B	8010-8878	869	CCGTTAACCACATCAAGTC	AGGGGAGTGTCTAACTGTTT
F12	NS5B	8621-9481	861	GACCTAGTAGTCATCTCAGAA	GGAACAGTTAGCTATGGAGTGTAC
F13	NS5B-3'NTR	9237-9648	412	ATCTCTTCAATTGGGCGGTGAAG	CTCTCTGCAGTCATGCGGCTCACGGACCTT
