# Supplementary material for: High-Resolution Functional Profiling of Hepatitis C Virus Genome
Source: PLoS Pathog. 2008 Oct 17;4(10):e1000182. doi: 10.1371/journal.ppat.1000182 (PMC2564836; doi:10.1371/journal.ppat.1000182)
Supplement: Table S2 — Primers used for Functional Profiling analysis of HCV genome (0.03 MB PDF) [file ppat.1000182.s010.pdf]

Table S2. Primers used for Functional Profiling analysis of HCV genome.

Primer Name	Primer Sequence (5'-3')	Fragment covered
JFH-20	GAC ACT CCG CCA TGA ATC ACT	F1
JFH-133	AGA GCC ATA GTG GTC TGC GGA	F1
JFH-394	CCC AGA AGA CGT TAA GTT C	F1
JFH-593	CTA TAT GGG AAT GAG GGA CT	F1
JFH-815	GAG GAC GGG GTT AAT TAT GCA A	F1, F2
JFH-1029	AGA GAG TGG GGA ATA CGT C	F2
JFH-1244	CAA GAA TGC AAT TGC TCC A	F2
JFH-1452	TCA TTG TCA TCC TTC TGC T	F2
JFH-1672	CAA CCG CTT TAA CTC GTC A	F2, F3
JFH-1858	CCC AGT GTA CTG TTT CAC	F3
JFH-2056	CAC CAG AGC TGA CTT CAA C	F3
JFH-2278	CGC ATG CAA CTT CAC TCG T	F3
JFH-2464	CCT CTC ACC TGC TAT CAC AA	F3, F4
JFH-2659	CAT CTT CTT CGT GGC AGC TT	F4
JFH-2876	TG TCT GTG GTG GTT GTG CT	F4
JFH-3068	TTG ACA CAT GTG CCG TAC TT	F4
JFH-3263	GTG GAA CCC ATC ATC TTC A	F4, F5
JFH-3431	GCT CCC ATC ACT GCT TAT	F5
JFH-3613	CAA GAC TCT AGC CGG CTT AC	F5
JFH-3819	GAC CCA TTT CGA CCT TGA AG	F5
JFH-4030	GTA CTT GCA TGC TCC AAC T	F5, F6
JFH-4233	CCA CAT ATG GCA AAT TTC TC	F6
JFH-4427	CAT CCC GAT ATA GAA GAG GT	F6
JFH-4620	TCT CCA TAA TAC CAG CTC AG	F6
JFH-4822	CAC AGG TAG AGG AAG ACA	F6, F7
JFH-5015	CTT GAA TTT TGG GAG GCA GT	F7
JFH-5216	ACA CCT CTC CTG TAC CGT T	F7
JFH-5417	AAC CAG CGA GTC GTC GTT	F7
JFH-5615	CCC AAA GTG GAA CAA TTT TG	F7, F8
JFH-5831	ACC GGC TTT GTC GTC AGT	F8
JFH-6040	CAT CTG CGC GGC CAT TCT	F8
JFH-6234	ACA ATT GGA TAA CTG AGG ACT	F8
JFH-6450	CCA ACA TCT CTG GCA ATG T	F8, F9
JFH-6641	GGG TCG TAC TCC TAT GTA AC	F9
JFH-6855	CAG ACG TAT TGA GGT CCA TG	F9
JFH-7025	GAC ATG GTC GAT GCC AAC	F9
JFH-7229	CTC GTG GAA TCG TGG AGG A	F9, F10
JFH-7416	GTG ATG CAG GCT CGT CCA	F10
JFH-7620	GGT CTT GGT CTA CTT GCT C	F10
JFH-7820	GTA ACT TTT GAC AGG ACG CA	F10
JFH-8010	CCG TTA ACC ACA TCA AGT C	F10, F11
JFH-8209	GCT TCC TCA GGC GGT AAT	F11
JFH-8418	CTG CCA TAC ACT CGC TGA	F11
JFH-8621	GAC CTA GTA GTC ATC TCA GAA	F11, F12
JFH-8822	AGA GAC CCA ACC ACT CCA	F12
JFH-9011	GGA TCA GTA TAC TCC GTG AA	F12
JFH-9237	ATC TCT TCA ATT GGG CGG TGA AG	F12, F13
JFH-9406	CGT AGG GGT AGG CCT CTT	F13
